# Maternal influenza and birth outcomes: systematic review of comparative studies

**DOI:** 10.1111/1471-0528.14143

**Published:** 2016-06-06

**Authors:** DB Fell, DA Savitz, MS Kramer, BD Gessner, MA Katz, M Knight, JM Luteijn, H Marshall, N Bhat, MG Gravett, B Skidmore, JR Ortiz

**Affiliations:** ^1^ Department of Epidemiology, Biostatistics and Occupational HealthMcGill UniversityMontrealQCCanada; ^2^ Better Outcomes Registry & Network (BORN)CHEO Research InstituteOttawaONCanada; ^3^ Department of EpidemiologyBrown UniversityProvidenceRIUSA; ^4^ Department of Obstetrics and GynecologyBrown UniversityProvidenceRIUSA; ^5^ Department of PediatricsMcGill University Faculty of MedicineMontrealQCCanada; ^6^ Agence de Médecine PréventiveParisFrance; ^7^ Independent ConsultantTel AvivIsrael; ^8^ National Perinatal Epidemiology UnitUniversity of OxfordOxfordUK; ^9^ Queen Mary University of LondonLondonUK; ^10^ Vaccinology and Immunology Research Trials UnitWomen's and Children's HospitalNorth AdelaideSAAustralia; ^11^ School of MedicineUniversity of AdelaideNorth AdelaideSAAustralia; ^12^ Robinson Research InstituteUniversity of AdelaideNorth AdelaideSAAustralia; ^13^ PATHSeattleWAUSA; ^14^ Department of Obstetrics and GynecologyUniversity of WashingtonSeattleWAUSA; ^15^ Global Alliance to Prevent Prematurity and StillbirthSeattle Children'sSeattleWAUSA; ^16^ Independent ConsultantOttawaONCanada; ^17^ Initiative for Vaccine ResearchWorld Health OrganizationGenevaSwitzerland

**Keywords:** Fetal death, influenza, pregnancy, preterm birth, small‐for‐gestational‐age birth, systematic review

## Abstract

**Background:**

Although pregnant women are considered at high risk for severe influenza disease, comparative studies of maternal influenza and birth outcomes have not been comprehensively summarised.

**Objective:**

To review comparative studies evaluating maternal influenza disease and birth outcomes.

**Search strategy:**

We searched bibliographic databases from inception to December 2014.

**Selection criteria:**

Studies of preterm birth, small‐for‐gestational‐age (SGA) birth or fetal death, comparing women with and without clinical influenza illness or laboratory‐confirmed influenza infection during pregnancy.

**Data collection and analysis:**

Two reviewers independently abstracted data and assessed study quality.

**Main results:**

Heterogeneity across 16 studies reporting preterm birth precluded meta‐analysis. In a subgroup of the highest‐quality studies, two reported significantly increased preterm birth (risk ratios (RR) from 2.4 to 4.0) following severe 2009 pandemic H1N1 (pH1N1) influenza illness, whereas those assessing mild‐to‐moderate pH1N1 or seasonal influenza found no association. Five studies of SGA birth showed no discernible patterns with respect to influenza disease severity (pooled odds ratio 1.24; 95% CI 0.96–1.59). Two fetal death studies were of sufficient quality and size to permit meaningful interpretation. Both reported an increased risk of fetal death following maternal pH1N1 disease (RR 1.9 for mild‐to‐moderate disease and 4.2 for severe disease).

**Conclusions:**

Comparative studies of preterm birth, SGA birth and fetal death following maternal influenza disease are limited in number and quality. An association between severe pH1N1 disease and preterm birth and fetal death was reported by several studies; however, these limited data do not permit firm conclusions on the magnitude of any association.

**Tweetable abstract:**

Comparative studies are limited in quality but suggest severe pandemic H1N1 influenza increases preterm birth.

## Tweetable abstract

Comparative studies are limited in quality but suggest severe pandemic H1N1 influenza increases preterm birth.

## Introduction

Pregnant women are considered vulnerable to serious influenza disease and related complications. On the basis of evidence documenting excess influenza‐related mortality in pregnant women during historical and recent pandemics^1^, ^2^, ^3^ and higher rates of influenza‐related morbidity requiring hospitalisation during seasonal epidemics,^4^, ^5^, ^6^ many countries advise that women who are, or will be, pregnant during the influenza season be immunised with inactivated influenza vaccine.^7^, ^8^, ^9^ Since 2012, the World Health Organization (WHO) has recommended that countries expanding or initiating influenza vaccination programs prioritise pregnant women for vaccine receipt.^10^ While the primary goal of these recommendations is to protect pregnant women from severe influenza disease, benefits of maternal immunisation have also been shown to extend to neonates through transfer of maternal antibodies, providing passive immunity against influenza virus infection.^11^

The possibility that maternal influenza immunisation could be of additional value beyond prevention of maternal and neonatal influenza illness is of considerable public health interest.^12^, ^13^, ^14^ In recent years, several observational studies and one secondary analysis of a randomised clinical trial have reported risk reductions for several adverse perinatal outcomes following influenza immunisation during pregnancy.^15^ The biologic plausibility of such findings depends on there being a direct or indirect adverse effect of maternal influenza disease on fetal health that is preventable via maternal immunisation. However, evidence on the effect of maternal influenza disease on birth outcomes is limited. The literature is dominated by descriptive case series reports that often lack complete information on birth outcomes,^16^ and existing systematic reviews have either predominantly synthesised evidence from descriptive studies of the 2009 H1N1 pandemic^16^, ^17^, ^18^ or focused on teratogenic effects of early pregnancy influenza virus infection.^19^

To address this evidence gap and inform expectations of possible benefits of maternal influenza vaccination on birth outcomes, the WHO initiated an evidence review of maternal influenza disease and adverse birth outcomes.^20^ The objective of this systematic review was to assess the risk of preterm birth, small‐for‐gestational‐age (SGA) birth and fetal death among women with clinical influenza disease and/or laboratory‐confirmed influenza virus infection during pregnancy, compared with women with no influenza during pregnancy.

## Methods

We developed a systematic review protocol (available on request) and prepared this manuscript following the Preferred Reporting Items for Systematic Reviews and Meta‐Analyses (PRISMA) recommendations.^21^

### Search strategy and study selection

This manuscript focuses on results from comparative studies; however, as part of a broader WHO evidence review,^20^ the literature searches and screening procedures pertain to the full evidence review. We performed electronic literature searches in MEDLINE, EMBASE, CINAHL and the Cochrane Library from inception to 5 December 2014. A medical librarian developed a sensitive search strategy utilising medical subject headings (e.g. ‘Influenza, Human’, ‘Pregnancy Complications’) and keywords (e.g. influenza, antenatal), and a second librarian peer‐reviewed the strategy^22^ (full search strategy provided in Appendix S1). Following de‐duplication, search records were uploaded into online software (ABSTRAKR^23^) and screened by two independent reviewers.

As part of the full WHO evidence review, we considered studies eligible for full‐text review if they (i) employed comparative designs (i.e. cohort, case‐control, cross‐sectional), descriptive designs (i.e. ecologic, case series, case report) or were systematic reviews; (ii) examined women with clinical influenza illness and/or laboratory‐confirmed influenza virus infection during pregnancy; and (iii) assessed any of the following primary outcomes: preterm birth (birth at less than 37 weeks of gestation), SGA birth (birthweight below the 10th percentile for gestational age and sex), fetal death (including miscarriage or stillbirth), or secondary outcomes: preterm birth utilising alternate gestational age thresholds, mean gestational age, low birthweight (<2500 g), mean birthweight. The preferred working definition for each outcome is shown in Table S1; however, individual studies were not required to exactly meet these definitions to be included. As we were not aware of any evidence concerning the most potentially susceptible time window for fetal exposure to maternal influenza in relation to our outcomes, we did not restrict the exposure definition to any particular gestational age range.

We made the following exclusions: non‐English language, editorials, commentaries, narrative reviews, clinical practice guidelines, conference abstracts or literature not in peer‐reviewed journals. The same reviewers independently evaluated the full text of all studies identified in the first stage of screening and resolved disagreements through consensus. Initially, we included influenza vaccination studies only if they evaluated our review outcomes relative to maternal influenza illness. Following completion of the initial screening process, however, we made a *post hoc* decision to include randomised controlled trials (RCTs) of influenza vaccination during pregnancy in narrative syntheses (but not quantitative meta‐analyses) if a placebo control group was used. In addition to providing estimates of vaccine efficacy, RCTs can help characterise the contribution of a vaccine‐preventable pathogen, such as influenza, to a range of clinical outcomes.^24^ Thus, we interpreted any differences in rates of adverse birth outcomes between vaccinated and unvaccinated women as attributable to differences in the contribution of influenza infection to the chain of events leading to the outcome.^24^

### Data extraction and quality assessment

We developed a data collection form to extract information on study design and population, inclusion and exclusion criteria, definition and ascertainment of exposure and outcomes, method of gestational age measurement and confounding variables considered in any analyses. For binary outcomes, we extracted raw cell counts to reconstruct 2 × 2 tables and any measures of effect (i.e. relative risks (RR), hazards ratios (HR), odds ratios (OR), or risk differences (RD)) with 95% confidence intervals (CI). Where possible, we calculated crude effect estimates using raw data from the reconstructed 2 × 2 tables when they were not reported by the study.

Individual study quality was assessed by two reviewers using the Newcastle‐Ottawa Scale for comparative observational studies^25^ or the Cochrane Collaboration risk of bias tool for RCTs.^26^ During our review, we developed a concern that ascertainment of clinical influenza disease and/or laboratory‐confirmed influenza virus infection may have been influenced by concern about the pregnancy (for example, there may have been a different threshold for influenza diagnosis/hospitalisation among women with high‐risk pregnancies or poor pregnancy outcome than among women with low‐risk pregnancies) resulting in differential misclassification of the exposure by study outcome. The impact of any such differential ascertainment would be expected to inflate the magnitude of effect estimates. To assess the risk of this potential differential exposure misclassification, which we denoted ‘diagnostic ascertainment bias’, we developed a rating system specifically for this review (Appendix S2) and rated each study as having low, medium, high or very high risk of diagnostic bias based on the method of influenza ascertainment. Using an adapted GRADE framework,^27^, ^28^ we rated the quality of evidence across studies for each primary outcome as high, moderate, low, or very low based on factors such as study design and limitations, inconsistency in study findings, and imprecision (Appendix S3).^27^, ^28^

### Data synthesis and analysis

We summarised study characteristics in descriptive tables and Forest plots. To determine whether meta‐analyses were appropriate, we considered clinical heterogeneity (i.e. measurement of influenza disease, clinical populations, outcome definitions and ascertainment), design heterogeneity (i.e. study design, analytical approach, extent of control for confounding factors), as well as statistical heterogeneity.^29^ The latter was quantified with the *I* ^*2*^ statistic, calculated using the natural log of the individual‐study adjusted effect estimates (or crude estimates when adjustment was not performed by the original study) and 95% CIs in a random‐effects model.^30^ The non‐central *χ* ^2^ method was used to compute a 95% CI around the *I* ^*2*^ statistic.^31^ Outcomes with *I* ^*2*^ values <75% were considered eligible for meta‐analysis if clinical and design characteristics were not qualitatively judged as too variable. Conversely, all outcomes with *I* ^*2*^ values ≥75% were considered inappropriate for meta‐analysis, regardless of other considerations. In such cases, we attempted to explore possible sources of heterogeneity in *post hoc* analyses by evaluating outcomes within more homogeneous subgroups, defined according to methodological quality and characteristics of influenza. We used STATA SE 12.1 software (Stata‐Corp LP, College Station, TX, USA) for all quantitative analyses.

## Results

### Study selection

**Figure 1:**
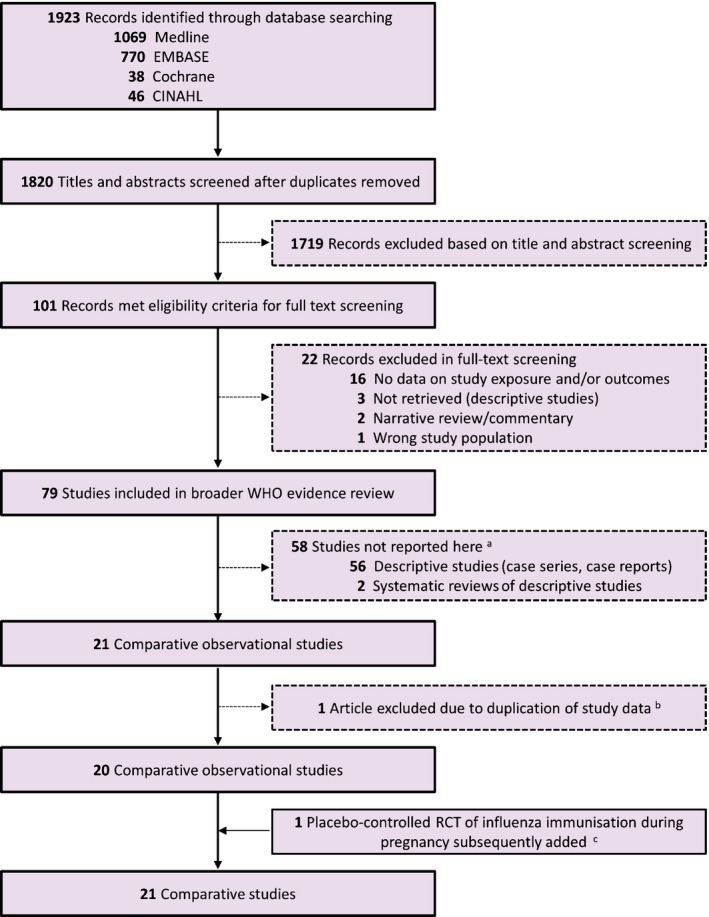
Preferred Reporting Items for Systematic Reviews and Meta‐Analyses (PRISMA) flow diagram showing study selection process. ^a^Descriptive studies and systematic reviews were screened as part of the overall evidence review, but are not reported in this publication. ^b^Yates et al. (2010)^32^ and Pierce et al. (2011)^33^ used the same study population, the former representing an earlier version of the study, published before full follow‐up had been completed. Only the Pierce et al. study^33^ is reported in this review. ^c^Subsequent to the original screening, a placebo‐controlled randomised clinical trial (RCT) of influenza immunisation during pregnancy was included.^11^

We identified 1923 records through electronic literature searches, of which 101 were eligible for full‐text review following title and abstract screening (Figure 1). Twenty‐two articles were excluded after full‐text review, leaving 79 studies in the broader WHO evidence review, including 56 descriptive studies and two systematic reviews of descriptive studies, not reported here. Among the comparative studies, we initially included 21 observational studies, but later excluded one^32^ after confirming that more complete follow‐up data on the same subjects had been reported in a subsequent publication.^33^ After completing our initial screening, we added one placebo‐controlled RCT of influenza immunisation during pregnancy, bringing the final number of comparative studies to 21.^11^

### Study characteristics and influenza ascertainment methods

Twelve studies were published in 2010 or later,^11^, ^33^, ^34^, ^35^, ^36^, ^37^, ^38^, ^39^, ^40^, ^41^, ^42^, ^43^ five between 2000 and 2009,^44^, ^45^, ^46^, ^47^, ^48^ and the remainder prior to 2000.^49^, ^50^, ^51^, ^52^ All of the observational studies originated from high‐income countries, including half from the USA;^34^, ^35^, ^36^, ^38^, ^40^, ^43^, ^44^, ^46^, ^50^, ^51^ the RCT was carried out in South Africa.^11^ Thirteen studies reported findings from pre‐2009 seasonal epidemics,^38^, ^41^, ^42^, ^43^, ^44^, ^45^, ^46^, ^47^, ^48^, ^49^, ^50^, ^51^, ^52^ five assessed influenza during the 2009 H1N1 pandemic,^33^, ^35^, ^36^, ^37^, ^39^ and remaining studies assessed a combination of seasonal influenza and 2009 pandemic H1N1 (pH1N1) influenza^34^, ^40^ or studied influenza seasons post‐2009.^11^ The majority of studies used either a retrospective^33^, ^35^, ^36^, ^38^, ^39^, ^40^, ^42^, ^44^, ^46^ or prospective^37^, ^41^, ^43^, ^47^, ^48^, ^49^, ^50^, ^51^ cohort design (Table S2).

With the exception of the four prospective seroepidemiological studies, in which paired maternal sera collected in early gestation and postpartum were tested for influenza virus infection during pregnancy^48^, ^49^, ^50^, ^51^ and the RCT with active surveillance,^11^ remaining studies ascertained influenza among pregnant women who presented for medical care with symptoms of clinical illness (Table S3). Four studies classified influenza status based on a self‐reported measure of influenza‐like illness collected either prospectively during pregnancy^41^, ^45^ or by post‐partum questionnaire.^34^, ^47^ Among 12 studies that ascertained influenza illness diagnosed during healthcare visits, there was substantial variation in the type of clinical settings (i.e. ambulatory versus hospitalisations), gestational timing of when diagnoses were ascertained (i.e. during health care visits at any gestational age versus only during hospitalisations in which the delivery occurred) and use of laboratory testing for diagnostic confirmation. We considered two studies to be at very high risk of diagnostic ascertainment bias owing to influenza ascertainment only at the time of the hospitalisation to give birth (i.e. coincident temporal diagnosis of influenza),^38^, ^44^ seven studies were considered to be at high risk,^33^, ^34^, ^35^, ^42^, ^46^, ^47^, ^52^ and the remaining studies at medium or low risk (Table S3).

### Preterm birth

**Figure 2:**
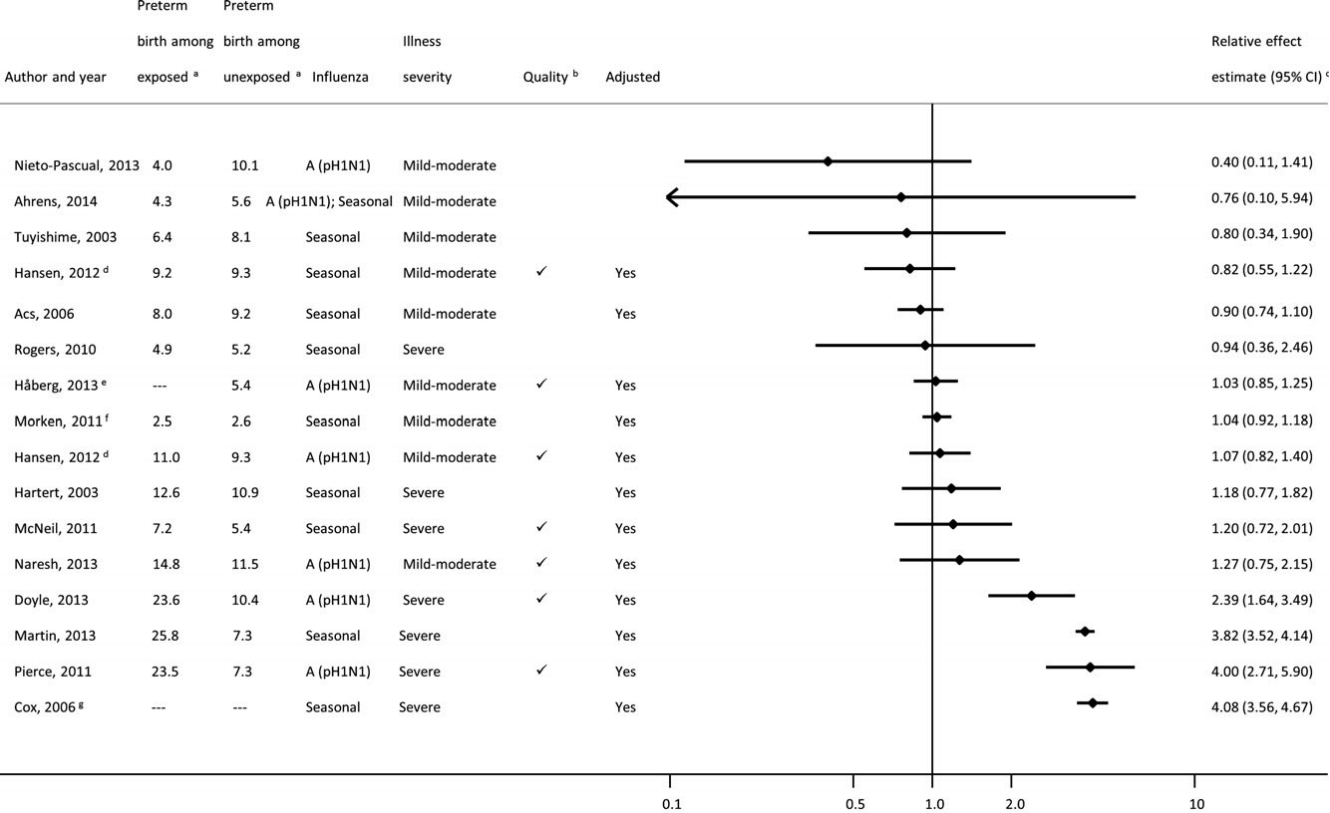
Forest plot of individual study results for association between influenza illness during pregnancy and preterm birth. Small, black diamond markers indicate individual study point estimate, with corresponding 95% confidence intervals (CIs) represented by horizontal bars. ^a^Risk of preterm birth per 100 women classified as having (exposed) or not having (unexposed) influenza illness/infection during pregnancy. ^b^For observational studies, ‘
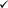
’ indicates a Newcastle‐Ottawa Score ≥8, risk of diagnostic ascertainment bias not rated as ‘very high’, and exposure not measured using self‐reported questionnaire. ^c^Crude estimates were used in place of adjusted estimates when the latter were not provided. ^d^Hansen (2012)^40^ is shown twice: one estimate for 2009 A (pH1N1) and one for 2008–2009 influenza season. ^e^Håberg (2013)^39^ did not provide the risk of preterm birth by exposure group. Overall risk in the study population was 5.4/100 singleton live births. ^f^Morken (2011)^41^ studied spontaneous preterm birth only. ^g^Baseline risk of preterm birth in the study population was not provided.^44^

Sixteen studies providing 17 estimates (one study provided separate estimates for two influenza seasons^40^) assessed preterm birth (Figure 2; Table S4). We did not compute a pooled estimate, primarily due to high statistical heterogeneity (*I* ^*2*^ = 98%; 95% CI 97–98); however, heterogeneity in influenza assessment methods was also a concern. Overall, individual‐study ratio estimates of effect displayed in Figure 2 ranged widely (from 0.40 to 4.08). Among the 13 adjusted estimates provided, nine had confidence intervals that included the null value (with point estimates ranging from 0.82 to 1.27) and four reported statistically significant estimates greater than one (with point estimates ranging from 2.39 to 4.08). Results from the RCT of influenza vaccination are not displayed in the Forest plot, but the risk ratio for preterm birth computed from raw study data did not indicate any difference in preterm birth risk between treatment arms (Table S4). Using a modified GRADE framework,^28^ we rated the quality of evidence across the 16 studies as very low (Appendix S3).

Aside from one study of spontaneous preterm birth,^41^ baseline risks of preterm birth across the study populations ranged from 5.2 to 11.5% among women with no influenza disease (Figure 2), comparable to population estimates for preterm birth in high‐income countries (Table S5). Among women with influenza disease, the range of preterm birth risks extended from 4.0 to 25.8%. Three of seven studies that ascertained only severe maternal influenza illness (i.e. all or most women were hospitalised, or selective laboratory testing was carried out only on women with symptoms of severe influenza disease) reported preterm birth risks in excess of 20% among women with influenza.^33^, ^35^, ^38^

After subgrouping studies according to similarity in methods of influenza assessment, methodological quality and influenza season characteristics (Table S6), statistical heterogeneity remained high (*I* ^*2*^ **> 80%) within most subgroups. Among the studies considered to be of highest quality (i.e. a Newcastle‐Ottawa Scale score ≥8, risk of diagnostic ascertainment bias not rated as ‘very high’, and exposure not measured using a self‐reported questionnaire), statistical heterogeneity was only marginally lower (*I* ^*2*^ = 89%; 95% CI 79–92). The only subgroup in which heterogeneity was substantially reduced was that containing only studies of mild‐to‐moderate influenza illness (*I* ^*2*^ **= 0%; 95% CI 0–53; Table S6).

Among six studies considered of highest methodological quality, two of severe 2009 pH1N1 influenza disease reported a preterm birth risk of 24% among women with influenza,^33^, ^35^ resulting in adjusted odds ratios of 2.4 and 4.0 (Figure S1; Table S6). Three other 2009 pH1N1 studies based on a wider range of illness severity reported no significantly elevated risk of preterm birth (adjusted odds ratios from 1.03 to 1.27).^36^, ^39^, ^40^ In the two highest‐quality studies from seasonal epidemic years, no association was observed between influenza and preterm birth, whether based on hospitalisation for influenza disease^42^ or broader ascertainment criteria.^40^ In the RCT of influenza vaccination (Table S4), there was no difference in the proportions of preterm birth in the two arms despite a 50% reduction in maternal influenza disease in the active treatment group.^11^

### SGA birth

Five studies providing six estimates assessed SGA birth.^36^, ^37^, ^40^, ^42^, ^48^ Three studies defined SGA birth as sex‐specific birthweight <10th percentile, each relative to different reference standards,^36^, ^40^, ^42^ another used birthweight <10th percentile determined from the distribution within the study population,^37^ and the fifth examined intrauterine growth restriction but provided no specific definition (Table S7).^48^ Baseline risks of SGA birth ranged from 3.9^48^ to 14.1%^37^ in women with no influenza disease during pregnancy, and from 2.8^48^ to 15.3%^42^ among women who had influenza disease. Aside from differences in definitions of SGA birth, quality was a lesser concern in this group of studies than it was for preterm birth – most of the point estimates originated from studies with a Newcastle‐Ottawa Score of 8 or 9 and most studies were considered to have a medium or low risk of diagnostic ascertainment bias. Using the modified GRADE framework,^28^ we rated the quality of evidence across the studies as low (Appendix S3).

**Figure 3:**
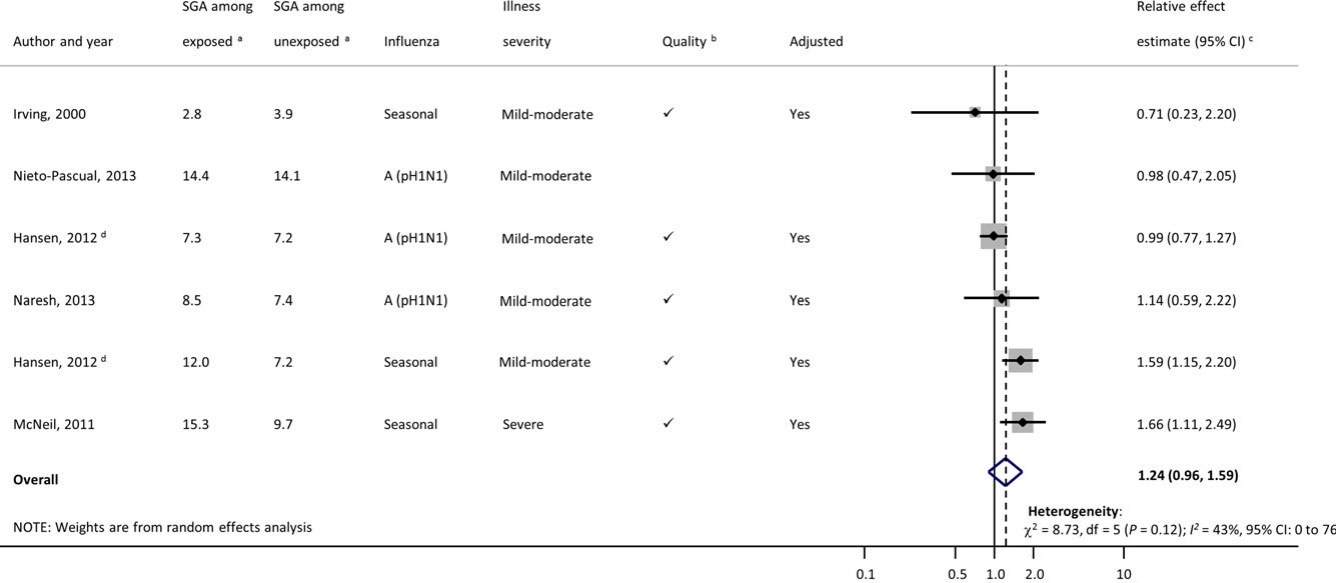
Forest plot of individual study results for association between influenza illness during pregnancy and small‐for‐gestational‐age (SGA) birth. Small, black diamond markers indicate individual study point estimate, with corresponding 95% confidence intervals (CIs) represented by horizontal bars. ^a^Risk of SGA birth per 100 women classified as having (exposed) or not having (unexposed) influenza illness/infection during pregnancy. ^b^’
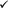
’ indicates a Newcastle‐Ottawa Score ≥8, risk of diagnostic ascertainment bias not rated as ‘very high’, and exposure not measured using self‐reported questionnaire. ^c^Crude estimates were used in place of adjusted estimates when the latter were not provided. ^d^Hansen (2012)^40^ is shown twice: one estimate for 2009 A (pH1N1) and one for 2008–2009 influenza season.

Among the five adjusted ratio estimates provided, three had confidence intervals that included the null value (with point estimates ranging from 0.71 to 1.14), and two reported statistically significant estimates greater than one (with point estimates of 1.59 and 1.66). As the *I* ^*2*^ statistic was 43% (95% CI 0–76) and clinical heterogeneity was not considered prohibitively high, we computed a pooled OR of 1.24 (95% CI 0.96–1.59; Figure 3). The number of studies examining this outcome was insufficient for subgroup analyses; however, we noted that both studies reporting an association between SGA birth and maternal influenza were from non‐pandemic influenza seasons, whereas none of the three studies from the 2009 H1N1 pandemic found any association. The only study of severe influenza disease reported an increased risk of SGA birth (adjusted OR 1.66; 95% CI 1.11–2.49).^42^ Another found no association with SGA birth in a primary analysis, but in a secondary analysis limited to women with severe influenza disease, reported an adjusted OR of 2.35 (95% CI 1.03–5.36).^36^

### Fetal death

**Figure 4:**
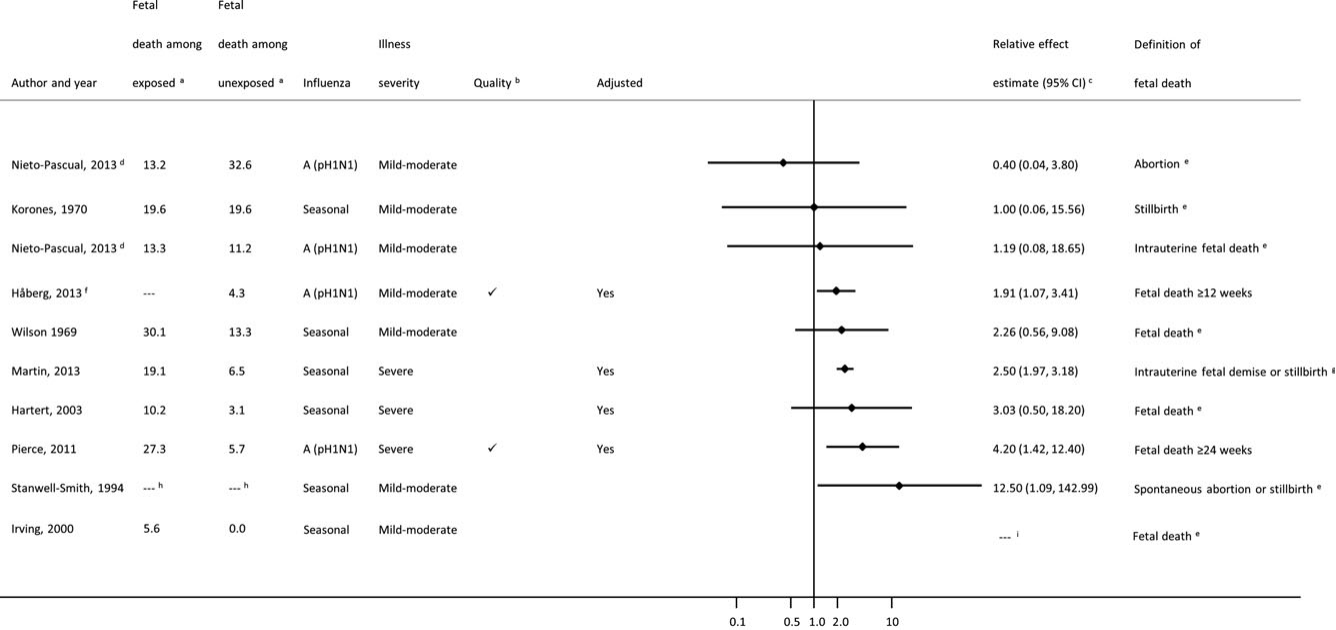
Forest plot of individual study results for association between influenza illness during pregnancy and fetal death. Small, black diamond markers indicate individual study point estimate, with corresponding 95% confidence intervals (CIs) represented by horizontal bars. ^a^Risk of fetal death birth per 1000 women classified as having (exposed) or not having (unexposed) influenza illness/infection during pregnancy. ^b^For observational studies, ‘
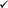
’ indicates a Newcastle‐Ottawa Score ≥8, risk of diagnostic ascertainment bias not rated as ‘very high’, and exposure not measured using self‐reported questionnaire. ^C^ Crude estimates were used in place of adjusted estimates when the latter were not provided. ^d^Nieto‐Pascual (2013)^37^ is shown twice: one estimate for abortion (RR: 0.40) and one for intrauterine fetal death (RR: 1.19). ^e^Not further defined. ^f^Håberg (2013)^39^ did not provide the risk of fetal death by exposure group. Overall risk in the study population was 4.3 fetal deaths per 1000 pregnancies. ^g^Based on ICD‐9 diagnostic codes. ^h^Risk of fetal death cannot be calculated because this was a case‐control study. ^i^Irving (2000)^48^ had no fetal death events among unexposed women and only one event among exposed women; therefore an effect estimate could not be computed.

Fetal death was reported in 10 publications (Figure 4; Table S8); however, meta‐analysis of results across studies was not possible because of high variability in fetal death definitions. Several studies used specific terminology to refer to mortality outcomes, such as spontaneous abortion or stillbirth, but did not specify the gestational age ranges underlying these definitions,^37^, ^38^, ^50^, ^52^ and three others provided no clarifying information.^46^, ^48^, ^51^ Only three of the studies specified a definition of fetal death.^11^, ^33^, ^39^ The GRADE quality of evidence rating^28^ across the studies was very low (Appendix S3).

Inadequate numbers of mortality events in most of these studies also seriously limited meaningful interpretation of results. Although there was a combined total of 103 902 fetal deaths reported from over 17 million participants, most (103 326/103 902; 99.4%) were reported from one very large study conducted in the USA across 10 influenza seasons using a hospitalisation database.^38^ Despite the large sample size, we considered this study to be at very high risk of diagnostic ascertainment bias, as influenza was ascertained only at the time of hospitalisation to give birth. The two highest‐quality studies were conducted during the 2009 pandemic and ascertained influenza disease occurring at any point during pregnancy. One reflected only severe maternal illness requiring hospitalisation (adjusted OR 4.2; 95% CI 1.4–12.4),^33^ whereas the other reflected mild‐to‐moderate maternal influenza illness severity and reported an adjusted hazard ratio of 1.91 (95% CI 1.07–3.41).^39^ In the South African RCT of influenza vaccination (not displayed in Figure 4; see Table S8), the number of miscarriages between 20 and 27 weeks and stillbirths ≥28 weeks was too small for meaningful interpretation (eight miscarriages and 24 stillbirths among non‐HIV infected women in total).^11^

Results for secondary outcomes are provided in Appendix S4 and Tables S9–S13.

## Discussion

### Main findings

Our systematic review has found that comparative studies of adverse birth outcomes following maternal influenza disease are limited in quantity and have produced inconsistent findings. The overall quality of evidence across studies was considered low to very low, due to the limited number of studies (particularly for SGA birth and fetal death), inconsistency of results and concerns about potential differential ascertainment of influenza by pregnancy outcome. Because of these limitations, firm conclusions are difficult to draw, although several studies suggest that severe maternal disease due to 2009 pH1N1 influenza is associated with preterm birth. We did not find evidence for an association with mild‐to‐moderate 2009 pH1N1 influenza, or with seasonal influenza of any severity. Drawing any overall conclusions on the risk of fetal death following maternal influenza is challenging due to insufficient mortality events in most studies and inconsistent study definitions of fetal death. The two highest‐quality studies both reported significantly increased risks of fetal death following maternal 2009 pH1N1 influenza illness, but high‐quality evidence from seasonal influenza time periods is lacking.

### Strengths and limitations

Strengths of our review include the comprehensive literature search strategy, focus on comparative studies and thorough assessment of the quality of the evidence. The most important limitation of the primary studies we reviewed relates to measurement and ascertainment of influenza. A particular concern was that seeking medical attention for influenza illness, being selected for microbiological confirmation or being hospitalised for influenza could have been motivated by concerns about the pregnancy,^53^ potentially leading to differential misclassification of the exposure by study outcome. The impact of any such differential ascertainment would be expected to exaggerate effect sizes, making influenza appear more strongly associated with adverse outcomes, though the potential extent of any such bias is unclear. There was also high potential for non‐differential misclassification of influenza status in many studies (e.g. if influenza illness was only ascertained in hospitalised cases or at a singular time point in gestation), which would tend to bias point estimates closer to the null value. With the exception of SGA birth, we were unable to pool data across studies due to high heterogeneity. We relied on the *I* ^*2*^ statistic to assess statistical heterogeneity; however, there were wide confidence intervals around many *I* ^*2*^ values as a result of the small number of studies.^54^ Many studies reported odds ratios rather than risk or hazard ratios, which may have inflated the apparent magnitude of any association in studies where rates of study outcomes exceeded 10%.^55^, ^56^ As noted by other systematic reviews,^15^, ^57^, ^58^ definitions of perinatal outcomes were heterogeneous and poorly reported. Methods of gestational age determination were also poorly reported despite being integral to defining outcomes such as preterm birth and fetal mortality.

### Interpretation

RCTs of influenza vaccination during pregnancy provide complementary evidence relating influenza disease and adverse birth outcomes, which is useful to consider in this context given the low quality, mixed evidence from epidemiologic studies. Two such trials have been published, both of which demonstrated efficacy in preventing influenza disease in pregnant women.^11^, ^59^ Results from the trials with respect to birth outcomes, however, were dissimilar. While the trial conducted in Bangladesh found higher mean birthweight and a lower percentage of SGA infants among a subset of infants born during the influenza season to influenza‐vaccinated women,^60^ the trial from South Africa did not detect any differences in preterm birth, low birthweight or median birthweight between treatment groups overall^11^, ^61^ or when assessed by maternal influenza infection status.^61^ Whether these divergent findings resulted from local differences in influenza season characteristics, vaccine components or comparators (active control^59^, ^60^ versus placebo control^11^) is unclear. Forthcoming evidence from two additional RCTs^62^, ^63^ may help clarify currently available results.

Most previous systematic reviews of influenza disease during pregnancy and birth outcomes have focused on descriptive studies from the 2009 H1N1 pandemic^16^, ^17^, ^18^ or on teratogenic effects of early pregnancy influenza virus infection.^19^ One other recent review included both descriptive and comparative studies, but provided limited synthesis of comparative findings for influenza disease and birth outcomes.^64^ Similar to our observations, most descriptive studies that support an association between pH1N1 influenza illness and adverse birth outcomes were of severely ill women,^16^, ^17^, ^18^, ^64^ while the few case series not reaching this conclusion described a broader population of pregnant women with milder clinical disease.^65^, ^66^ It remains unclear whether the associations with adverse birth outcomes found by some comparative studies from the 2009 pandemic reflect an increased biological susceptibility of pregnant women to the 2009 pH1N1 virus, possibly from lower levels of pre‐existing immunity or differences in virulence,^67^ or whether enhanced surveillance and potential for disproportionate diagnosis of pregnant women during that time period played a role.^36^, ^65^ As the epidemiological characteristics of the 2009 influenza pandemic were different from surrounding seasonal epidemics,^68^ a distinctive impact of the 2009 pH1N1 virus on pregnant women is plausible, but drawing a firm conclusion is limited by the small number of studies and the low quality of the comparative evidence.

Although sequelae from infection with some viral pathogens in pregnancy are well understood (e.g. congenital cytomegalovirus, rubella, varicella),^69^, ^70^ potential pathogenic effects of influenza viruses on the fetus are not. As the influenza virus is rarely transmitted across the placenta,^48^, ^71^ influenza virus infection is more likely to be associated with adverse birth outcomes through other mechanisms such as maternal fever and inflammation.^71^, ^72^, ^73^ Immunological responses, such as elevated pro‐inflammatory cytokine levels,^74^ can also influence placental function^75^, ^76^ and are recognised as an important pathway to preterm birth.^77^, ^78^ Secondary pneumonia was also identified as a contributing factor to excess fetal deaths during the influenza pandemic of 1918–1919.^1^ More recently, women hospitalised with 2009 pH1N1 influenza illness had a significantly increased risk of preterm delivery if they developed secondary pneumonia compared with those who did not develop pneumonia (71 versus 27%).^33^ In the case of an outcome such as preterm birth, which is defined only by the timing of birth, not by a clinical phenotype,^79^, ^80^ it is pertinent to note that biological mechanisms can be implicated not only for spontaneous preterm birth, but also for iatrogenic preterm birth, as the latter is often motivated by poor maternal condition.^33^, ^81^

## Conclusions

High‐quality data on the effect of maternal influenza disease on birth outcomes are necessary for informing public health policies for pregnant women and for clarifying expectations for improved perinatal outcomes following maternal influenza immunisation. Yet, our systematic review has found the evidence base from comparative studies on this subject to be limited. Although a small subgroup of higher‐quality studies found that severe pH1N1 influenza disease during pregnancy increased the risk of preterm birth and fetal death, there was little evidence that mild 2009 pH1N1 influenza, seasonal influenza disease of any severity, or subclinical infection in pregnant women was associated with the outcomes assessed in this review. A number of substantive gaps in the primary literature remain, including insufficient evidence on seasonal influenza disease, gestational timing of influenza disease, and evidence from low‐resource settings.

### Disclosure of interests

Full disclosure of interests available to view online as supporting information.

### Contribution to authorship

DBF developed the study protocol, screened articles for inclusion, extracted data from the included studies, analysed the data, interpreted the results and drafted the manuscript. DAS, MSK, JRO and BDG developed the study protocol, interpreted the results, and critically reviewed all drafts of the manuscript. MAK screened articles for inclusion, interpreted the results, and critically reviewed all drafts of the manuscript. MK, JML, HM, NB and MGG interpreted the results and critically reviewed all drafts of the manuscript. BS developed the search strategy, conducted the literature search, and critically reviewed all drafts of the manuscript. All authors have seen and approved the final version of this manuscript.

### Details of ethical approval

No ethical approval was required for this review as all data were already published in peer‐reviewed journals.

### Funding

This study was funded by a grant from the World Health Organization’s Initiative for Vaccine Research. The authors would like to acknowledge the contributions of the Centers for Disease Control and Prevention (CDC), which provides financial support to the World Health Organization Initiative for Vaccine Research (U50 CK000431).

### Disclaimer

The authors alone are responsible for the views expressed in this publication and they do not necessarily represent the decisions or policies of the World Health Organization.

## Supporting information


**Figure S1**. Forest plot of highest quality studies reporting preterm birth by influenza season and severity of maternal illness.

**Table S1**. Working definitions of primary and secondary review outcomes
**Table S2.** Descriptive characteristics of individual studies meeting inclusion criteria
**Table S3.** Methods used to ascertain clinical influenza and/or laboratory‐confirmed influenza virus infection during pregnancy
**Table S4.** Results of studies reporting preterm birth <37 weeks
**Table S5.** Publicly‐reported baseline rates of pregnancy outcomes in four high‐resource countries
**Table S6.** Assessment of heterogeneity among subgroups of studies reporting preterm birth <37 weeks
**Table S7.** Results of studies reporting small‐for‐gestational‐age (SGA) birth
**Table S8.** Results of studies reporting fetal death outcomes
**Table S9.** Results of studies reporting preterm birth <32 weeks
**Table S10.** Results of studies reporting preterm birth using other gestational age thresholds
**Table S11.** Results of studies reporting mean gestational age
**Table S12.** Results of studies reporting low birthweight (<2,500 grams), by method of accounting for gestational age
**Table S13.** Results of studies reporting mean birthweight, by method of accounting for gestational age

**Appendix S1**. Full search strategy with results

**Appendix S2**. Risk of diagnostic ascertainment bias assessment

**Appendix S3**. Assessment of primary study outcomes using adaptation of Grading of Recommendations Assessment, Development and Evaluation (GRADE) framework for assessing the quality of the evidence across studies

**Appendix S4**. Secondary outcomes
