# Supplementary material for: Maternal influenza and birth outcomes: systematic review of comparative studies
Source: BJOG. 2016 Jun 6;124(1):48–59. doi: 10.1111/1471-0528.14143 (PMC5216449; doi:10.1111/1471-0528.14143)
Supplement: Supplementary file 1 — Figure S1. Forest plot of highest quality studies reporting preterm birth by influenza season and severity of maternal illness. [file BJO-124-48-s001.pdf]

Figure S1. Forest plot of highest quality studies reporting preterm birth by influenza season and severity of maternal illness. Small, black diamond markers indicate individual study point, with corresponding 95% confidence intervals (CIs) represented by horizontal bars.

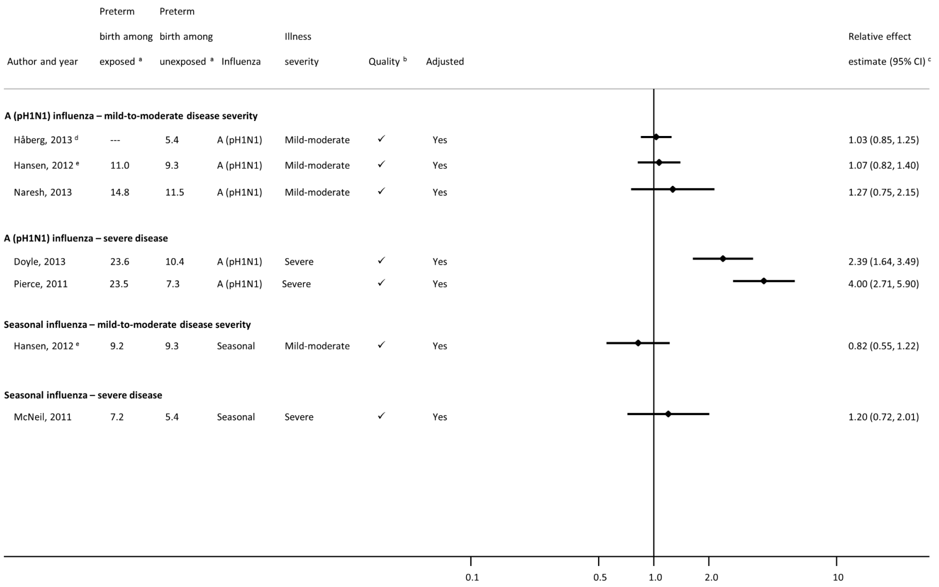

[a] Risk of preterm birth per 100 women classified as having (exposed) or not having (unexposed) influenza illness/infection during pregnancy.
[b] For observational studies, '✓' indicates a Newcastle Ottawa Score ≥8, risk of diagnostic ascertainment bias not rated as 'very high', and exposure not measured using self-reported questionnaire.
[c] Crude estimates were used in place of adjusted estimates when the latter were not provided.
[d] Håberg, 2013[39] did not provide the risk of preterm birth by exposure group. Overall risk in the study population was 5.4 per 100 singleton live births.
[e] Hansen, 2012[40] is shown twice: one estimate for 2009 A (pH1N1) and one for 2008–2009 influenza season.
