# Supplementary material for: Maternal influenza and birth outcomes: systematic review of comparative studies
Source: BJOG. 2016 Jun 6;124(1):48–59. doi: 10.1111/1471-0528.14143 (PMC5216449; doi:10.1111/1471-0528.14143)
Supplement: Supplementary file 2 — Table S1. Working definitions of primary and secondary review outcomes Table S2. Descriptive characteristics of individual studies meeting inclusion criteria Table S3. Methods used to ascertain clinical influenza and/or laboratory‐confirmed influenza virus infection during pregnancy Table S4. Results of studies reporting preterm birth <37 weeks Table S5. Publicly‐reported baseline rates of pregnancy outcomes in four high‐resource countries Table S6. Assessment of heterogeneity among subgroups of studies reporting preterm birth <37 weeks Table S7. Results of studies reporting small‐for‐gestational‐age (SGA) birth Table S8. Results of studies reporting fetal death outcomes Table S9. Results of studies reporting preterm birth <32 weeks Table S10. Results of studies reporting preterm birth using other gestational age thresholds Table S11. Results of studies reporting mean gestational age Table S12. Results of studies reporting low birthweight (<2,500 grams), by method of accounting for gestational age Table S13. Results of studies reporting mean birthweight, by method of accounting for gestational age [file BJO-124-48-s002.pdf]

Table S1. Working definitions of primary and secondary review outcomes [a]

Outcomes	Definition
**Primary outcomes**	
Preterm birth	Birth at less than 37 completed weeks of gestation
Small-for-gestational-age (SGA) birth [b]	Birth weight below the 10th percentile cut-off for gestational age and sex [c]
Fetal death [d]	Spontaneous antepartum or intrapartum death of a fetus at any gestation
Miscarriage	Spontaneous fetal death in early gestation, usually before week 28 completed weeks of gestation
Stillbirth	Spontaneous fetal death in mid-to-late gestation, usually at 20 to 28 completed weeks of gestation or later
**Secondary outcomes**	
Very preterm birth	Birth at less than 32 completed weeks of gestation
Mean gestational age	Average gestational age in completed weeks
Low birth weight	Birth weight less than 2,500 grams
Mean birth weight	Average birth weight in grams

[a] These were the working definitions used by the review; however individual studies were not required to exactly meet these definitions. Study-specific definitions are provided in the Supporting Information.

[b] Also denoted intrauterine growth restriction by some studies.

[c] The 10th percentile was defined using the study population by some studies, and using published reference standards by others (S7 Table). We also included studies of "fetal growth restriction" and "intrauterine growth restriction", since these terms are often used interchangeably with SGA birth in the literature.

[d] We used the term fetal death broadly to denote death of a fetus at any gestational age, and miscarriage or stillbirth, respectively, when specifically referring to fetal death in early or later stages of gestation. The WHO defines stillbirth as a fetal death at 28 weeks or later, but definitions vary between studies.

Table S2. Descriptive characteristics of individual studies meeting inclusion criteria

First author and year of publication	Study design	Location of study	Study population	Number of subjects	Study period	Outcomes relevant to this review
Madhi 2014 [1]	Randomized placebo-controlled clinical trial of trivalent inactivated influenza vaccine [a]	Soweto, South Africa	HIV-uninfected pregnant women were recruited from 4 antenatal clinics in Soweto [b]	2,116 HIV-uninfected pregnant women [b]	2011 influenza season (women recruited between March 3, 2011 and August 4, 2011) and 2012 influenza season (women recruited between March 6, 2012 and July 2, 2012)	▪ Preterm birth (<37 weeks)<br>▪ Low birth weight (<2,500 grams)<br>▪ Median birth weight<br>▪ Miscarriage (20–27 weeks)<br>▪ Stillbirth (≥28 weeks)
Ahrens 2014 [2]	Population-based case-control study (only the control series was included)	United States (Philadelphia, San Diego, New Hampshire, Rhode Island, New York State)	Participants of ongoing birth defect study (only non-malformed live born infants were included)	1,619 women	2006–2007 to 2009–2010 [c]	▪ Preterm birth (<37 weeks)
Doyle 2013 [3] [d]	Retrospective cohort study	Florida, United States	All live births in Florida	Primary analysis: 295,938 women (187 exposed women and 295,747 unexposed women)<br><br>Alternative comparison with pre-pandemic unexposed group: 23,866 women (187 exposed woman and 23,679 unexposed women) [e]	Primary analysis: all live births between June 1, 2009 and September 1, 2010<br><br>Alternative comparison with pre-pandemic unexposed group: all live births between January 1 and February 28,2009 [e]	▪ Preterm birth (<37 weeks)<br>▪ Very preterm birth (<32 weeks)<br>▪ Preterm birth <28 weeks<br>▪ Mean gestational age<br>▪ Low birth weight (<2,500 grams)<br>▪ Very low birth weight (<1,500 grams)<br>▪ Birth weight <1,000 grams<br>▪ Mean birth weight
Naresh 2013 [4]	Multicenter cohort study	United States (Pittsburgh, Denver, Seattle)	Exposed cohort: all inpatient and outpatient pregnant and postpartum	852 women	Pittsburgh: between September 2009 and May 2010	▪ Preterm birth (<37 weeks)<br>▪ Mean gestational age

			women within 6 weeks of delivery who tested positive for H1N1 influenza<br><br>Unexposed cohort: women who delivered at the same hospital with an expected date of delivery in the same calendar month (sampled at a ratio of 1 exposed: 5 unexposed)		Washington: between July 2009 and January 2010<br><br>Denver: between November 3, 2009 and May 3, 2010	▪ Small for gestational age<br>▪ Mean birth weight
Nieto-Pascual 2013 [5]	Cohort study (test-negative design)	Córdoba, Spain	Pregnant women who visited the obstetrics emergency unit at Reina Sofia University Hospital for suspected influenza	168 women	Pregnant women with a visit to the obstetrical emergency unit for suspected influenza between September 2009 and February 2010 were included and followed to delivery	▪ Preterm birth (<37 weeks)<br>▪ Mean gestational age<br>▪ Birth weight <10th percentile<br>▪ Mean birth weight<br>▪ Abortion<br>▪ Intrauterine fetal death
Martin 2013 [6]	Retrospective cohort study	United States	All pregnancy hospitalizations (antenatal undelivered admissions and delivery admissions)	17,548,022 total pregnancy hospitalizations [f]	Pregnancy hospitalizations with a date of discharge between 1998 and 2008	▪ Preterm birth (<37 weeks)<br>▪ Intrauterine fetal demise/stillbirth
Håberg 2013 [7]	Retrospective cohort study	Norway	All singleton pregnancies	113,331 women	Births in 2009 and 2010 (H1N1 pandemic)	▪ Preterm birth (<37 weeks)<br>▪ Term low birth weight (<2,500 grams)<br>▪ Fetal death
Hansen 2012 [8]	Retrospective cohort study	5 geographic regions in the United States (Northern and Southern California, Colorado, Georgia, and Mid-Atlantic States)	All live births to women continuously enrolled with Kaiser Permanente managed care consortium throughout their pregnancy (gaps of up to 45 days were allowed)	111,158 live births (109,015 pregnancies; 107,889 women)	All live births between July 1, 2008 to May 31, 2010	▪ Preterm birth (<37 weeks)<br>▪ Small for gestational age<br>▪ Low birth weight (<2,500 grams)

Morken 2011 [9]	Prospective cohort study	Norway	Target population: pregnant women recruited at 17–18 weeks' gestation<br><br>Study population: singleton pregnancies; excluded late abortions (<22 gestational weeks), congenital malformations, intrauterine fetal death and unknown type of delivery onset	67,310 for assessment of exposure at <13 weeks<br><br>60,689 for evaluation of exposure at ≥13 weeks<br><br>Participation rate was 43.5%	Pregnant women recruited between 1999 and 2008	▪ Spontaneous preterm birth (<37 weeks)
Pierce 2011 [10 d]	Retrospective matched cohort study	United Kingdom	Exposed cohort: 256 women admitted to hospital with lab-confirmed A/H1N1 influenza infection in pregnancy<br><br>Unexposed cohort: Sample of 1,220 pregnant women who delivered prior to the 2009 A/H1N1 pandemic	1,476 women	Exposed cohort: hospital admissions for A/H1N1 during pregnancy between September 1, 2009 and January 31, 2010 (A/H1N1 pandemic)<br><br>Unexposed cohort: deliveries between February 2005 and February 2006<br><br>National data: births in 2008	▪ Preterm birth (<37 weeks)<br>▪ Very preterm birth (<32 weeks)<br>▪ Low birth weight (<2,500 grams)<br>▪ Very low birth weight (<1,500 grams)<br>▪ Mean birth weight<br>▪ Fetal death
McNeil 2011 [11]	Retrospective cohort study	Nova Scotia, Canada	All singleton live births	132,588 births	Births between 1990 and 2002	▪ Preterm birth (<37 weeks)<br>▪ Small for gestational age<br>▪ Low birth weight (<2,500 grams)<br>▪ Mean birth weight<br>▪ Mean birth weight among term infants
Rogers 2010 [12]	Hospital-based prospective cohort study	Dallas, Texas, United States	Exposed women: laboratory-confirmed influenza infection during pregnancy	107 women had a lab-confirmed diagnosis of influenza, but only 81 (76%) had	Exposed cohort: pregnant women meeting the exposure definition with a visit to the labour and delivery triage area,	▪ Preterm birth (<37 weeks)<br>▪ Very preterm birth (<34 weeks)<br>▪ Mean gestational

			Unexposed women: all other pregnant women who gave birth at the same hospital in 2003 or 2004	complete follow-up of pregnancy outcomes<br><br>30,983 women who did not present with symptoms of influenza during pregnancy were considered unexposed	obstetrics and gynecology emergency room, prenatal clinics for high-risk obstetrical patients, or postpartum wards for suspected influenza between October 22, 2003, and January 18, 2004<br><br>Unexposed cohort: all deliveries in the same hospital in 2003 and 2004 not meeting the exposure definition	age<br>▪ Low birth weight (<2,500 grams)<br>▪ Mean birth weight
Cox 2006 [13]	Retrospective cohort study	United States	All pregnancy hospitalizations (antenatal undelivered admissions and delivery admissions)	6,277,508 total pregnancy hospitalizations [f]	Pregnancy hospitalizations with a date of discharge between 1998 and 2002	▪ Preterm birth (<37 weeks)
Acs 2006 [14]	Population-based case-control study (only the control series was included)	Hungary	Participants of ongoing birth defect study (only non-malformed live born infants sampled from the National Birth Registry were included)	38,151 births	Births between 1980 and 1996	▪ Preterm birth (<37 weeks)<br>▪ Mean gestational age<br>▪ Low birth weight (<2,500 grams)<br>▪ Mean birth weight
Hartert 2003 [15]	Retrospective matched cohort study	Tennessee, United States	Exposed women: pregnant women from a Tennessee Medicaid population with a respiratory hospitalization during influenza season<br><br>Unexposed women: women from the same Medicaid population matched by age, race, gestational week of pregnancy on the date corresponding	887 (293 exposed women and 587 matched unexposed women)	1985–1993	▪ Preterm birth (<37 weeks)<br>▪ Low birth weight (<2,500 grams)<br>▪ Very low birth weight (<1,500 grams)<br>▪ Mean birth weight<br>▪ Fetal death

			to the admission of the matched exposed woman, and presence or absence of high-risk medical conditions, but without a respiratory hospitalization during influenza season			
Tuyishime 2003 [16]	Prospective cohort study	Sherbrooke, Québec, Canada	Target population: all women who delivered at the Sherbrooke University Hospital Centre	517 women<br><br>Participation rate was 96%	All women who delivered between February 15, 2002 and April 30, 2002	▪ Premature birth<br>▪ Mean gestational age<br>▪ Low birth weight<br>▪ Mean birth weight
Irving 2000 [17]	Matched cohort study (prospective seroepidemiological study)	Nottingham, United Kingdom	Target population: women who delivered at University and City Hospitals, Nottingham	361 women with maternal ante- and postnatal sera, and cord sera (181 exposed women and 180 matched unexposed women)	Deliveries between May 1993 and July 1994	▪ Preterm birth [g]<br>▪ Intrauterine growth restriction [h]<br>▪ Mean birth weight<br>▪ Fetal death
Stanwell-Smith 1994 [18]	Case-control study	London, United Kingdom	Target population: pregnant women receiving care at one hospital	21 women (12 case women with a recent spontaneous abortion or stillbirth and 9 control women who had recently had a live birth)	Cases and controls had a last menstrual period on or after April 1, 1985.<br><br>Cases had a spontaneous abortion or stillbirth after January 1, 1986.	▪ Fetal death
Griffiths 1980 [19]	Matched cohort study (prospective seroepidemiological study)	London, United Kingdom	Target population: pregnant women receiving care at St. Bartholomew's Hospital during the study period	154 women with maternal ante- and postnatal sera (77 exposed women and 77 matched unexposed women)	Women receiving care at the study hospital with a last menstrual period between May 1975 and November 1975, December 1976 to June 1977, and August 1977 to April 1978 (3 consecutive influenza seasons)	▪ Mean birth weight
Korones 1970 [20] [i]	Matched cohort study (prospective	United States (3 collaborating	The sample for this study originated from	102 women were included in the	Women who were registered from October	▪ Mean birth weight<br>▪ Fetal death

	seroepidemiological study)	hospitals participating in the Collaborative Perinatal Study: Pennsylvania Hospital, Children's Hospital of Philadelphia, University of Tennessee)	the Collaborative Perinatal Study, comprising 12 participating academic centres in the United States and over 60,000 participants	analysis of influenza exposure (51 exposed women and 51 matched unexposed women)[f]	1959 through December 1963, at 3 of the collaborating institutions	
Wilson 1969 [21] [j]	Cohort study (prospective seroepidemiological study)	Los Angeles, California, United States	Pregnant women receiving care at the Los Angeles County-University of Southern California Medical Center, with serologic testing for Asian influenza during the latter part of pregnancy	487 women (299 exposed women and 188 unexposed women)	Women with a last menstrual period during the three month period in 1957 when epidemic Asian influenza circulated in Los Angeles	▪ Low birth weight (<2,500 grams)<br>▪ Fetal death

[a] 194 HIV-infected pregnant women were also recruited into the RCT, but only data from the HIV-uninfected cohort have been extracted for this review.

[b] Due to randomized assignment of influenza vaccination and the use of a placebo control group, we interpreted any differences in rates of adverse pregnancy outcomes as attributable to differences in influenza disease between vaccinated and unvaccinated pregnant women.

[c] The full study period included 2006–2007 to 2009–2010; however, data on influenza-like illness was only collected in 2009–2010.

[d] Additional clinical details for infected pregnant women are provided in the manuscript.

[e] Although an alternative pre-pandemic comparison group was assembled, no quantitative data were reported for this secondary comparison.

[f] The unit of analysis was the hospitalization (not unique pregnancies or unique women).

[g] Although preterm birth was listed as an outcome by the authors, it was not included in the assessment of preterm birth in this review since the authors matched exposed and unexposed subjects on gestational age.

[h] Intrauterine growth restriction was not defined by the authors.

[i] This study additionally assessed other viral pathogens. The number reported in this table only pertains to the number of subjects included in analyses of influenza exposure, not the number of women included in analyses of other viral exposures.

[j] The text also reported no difference in “intrauterine growth retardation” and “prematurity” between the exposure groups; however, no numerical information was provided.

Table S3. Methods used to ascertain clinical influenza and/or laboratory-confirmed influenza virus infection during pregnancy [a]

First author and year of publication	Type of influenza	Ascertainment of influenza at any gestation	Represents only severe influenza disease [b]	Influenza diagnoses only ascertained during hospitalizations	Clinical influenza and/or laboratory-confirmed influenza virus infection during pregnancy % (n/N)	Risk of diagnostic ascertainment bias [c]
**Active surveillance with laboratory confirmation**						
Madhi 2014 [11]	Seasonal	Yes [d]	No	No	2.8% (57/2,049) [e]	Low
**Medically attended influenza with laboratory confirmation** [33,35–37,43,52]						
Doyle 2013 [35]	2009 A (pH1N1)	Yes	Yes	Yes	0.06% (187/295,938)	High
Naresh 2013 [36]	2009 A (pH1N1)	Yes	No	No	n=142 [f]	Medium
Nieto-Pascual 2013 [37]	2009 A (pH1N1)	Yes	Yes	No	n=76 [f]	Medium
Pierce 2011 [33]	2009 A (pH1N1)	Yes	Yes	Yes	0.08%(256/314,135) [g]	High
Rogers 2010 [43]	Seasonal	Yes	No	No	0.3% (81/30,983)	Medium
Stanwell-Smith 1994 [52]	Seasonal	No	No	No	--- [h]	High
**Medically attended influenza identified using diagnostic codes** [38–40,42,44,46]						
Martin 2013 [38]	Seasonal	No [i]	Yes	Yes	0.3% (56,337/17,548,022) [j]	Very high
Håberg 2013 [39]	2009 A (pH1N1)	Yes	No	No	2% (2,278/113,331)	Medium
Hansen 2012 [40]	Seasonal and 2009 A (pH1N1)	Yes	No	No	Seasonal: 0.3% (368/109,015)<br>2009 A (pH1N1): 0.9% (959/109,015)	Medium
McNeil 2011 [42]	Seasonal	Yes [k]	Yes	Yes	0.2% (208/132,588)	High
Cox 2006 [44]	Seasonal	No [i]	Yes	Yes	0.3% (21,447/6,277,508) [j]	Very high
Hartert 2003 [46]	Seasonal	Yes	Yes	Yes	n=293 [f]	High
**Prospective serological testing of paired antenatal and postpartum maternal sera, irrespective of influenza illness** [48–51]						
Irving 2000 [80]	Seasonal	Yes	No	No	11% (182/1,659)	Low
Griffiths 1980 [49]	Seasonal	Yes	No	No	5.0% (79/1,595)	Low
Korones 1970 [50]	Seasonal	Yes	No	No	1.1% (52/4,930)	Low

Wilson 1969 [51]	Seasonal	Yes	No	No	n=299 [f]	Low
**Self-reported illness collected prospectively via antenatal questionnaire [41,45]**						
Morken 2011 [41]	Seasonal	Yes	No	No	54% [l]	Medium
Acs 2006 [45]	Seasonal	Yes	No	No	4.8% (1,838/38,151)	Medium
**Self-reported illness collected retrospectively via postnatal questionnaire [34,47]**						
Ahrens 2014 [34]	Seasonal and 2009 A (pH1N1)	Yes	No	No	6.1% (23/378)	High
Tuyishime 2003 [47]	Seasonal	Yes	No	No	64% (331/517)	High

[a] Studies that used more than one method to ascertain influenza have been categorized according to the most predominant method used.

[b] All or most women were hospitalized, laboratory testing was selectively carried out only on women with clinical symptoms of severe illness, or the study specified that only severe influenza cases were assessed.

[c] Risk for diagnostic ascertainment bias in each study was assessed using a rating system specifically developed for this review; see description in Appendix S2.

[d] Active surveillance for influenza virus infection from the time of enrollment at 20 to 36 weeks of gestational age.

[e] Rate of laboratory-confirmed influenza in the intervention and control arm, combined, for HIV-uninfected women (intervention arm: 1.8% [19/1,026]; control arm: 3.6 [38/1,023]).

[f] Since the comparison group of uninfected women was deliberately sampled at a predetermined ratio, the incidence of influenza cannot be computed.

[g] The denominator for this rate was published in a related earlier study.[32]

[h] Case-control study, therefore the incidence of influenza cannot be computed.

[i] Influenza hospitalizations throughout pregnancy were included in the study, but only those coincident with the timing of delivery were used in analyses of birth outcomes (i.e., women with antenatal hospitalizations for influenza earlier in pregnancy were not classified as having influenza).

[j] Represents the total number of influenza hospitalizations among pregnant women; however, only a subset of influenza hospitalizations (those coincident with the timing of delivery) were used in analyses of birth outcomes.

[k] Women with an influenza hospitalization during which the delivery also occurred were not classified as having influenza.

[l] Estimated from data presented in Table 4 of the original study for self-reported influenza/common cold.

Table S4. Results of studies reporting preterm birth <37 weeks

First author and year of publication	Definition	Risk of preterm birth <37 weeks, by influenza infection/illness (n/N) [a]	Type of effect measure reported	Crude effect measure (95% CI)	Adjusted effect measure (95% CI)	Adjustment methods (i.e., matching, multivariable adjustment, propensity scores) and adjustment variables	Newcastle Ottawa Scale [b] Selection	Compar-ability	Outcome/ Exposure
Madhi 2014 [1]	Live birth before 37 completed weeks of gestation	Exposed: 9.4 per 100 live births (96/1,023)[c]<br><br>Unexposed: 10.5 per 100 live births (108/1,026)[c]	RR	n/a due to randomization	0.89 (0.68–1.17) [d]	Randomization	--- [e]	--- [e]	--- [e]
Ahrens 2014 [2]	Live birth before 37 completed weeks of gestation	Exposed: 4.3 per 100 live births (1/23)<br><br>Unexposed: 5.6 per 100 live births (20/355)	OR	0.76 (0.10–5.94) [d]	Not provided	No adjustment	3	0	3
Doyle 2013 [3]	Live birth before 37 completed weeks of gestation	Exposed: 23.6 per 100 live births (45/191)<br><br>Unexposed: 10.4 per 100 live births (31,259/299,885)	OR	2.65 (1.90–3.70)	2.39 (1.64–3.49) [f]	Multivariable adjustment for maternal age, maternal race, maternal ethnicity, maternal education, maternal marital status, plurality, infant sex, tobacco use during pregnancy, alcohol use during pregnancy, previous preterm delivery, previous poor pregnancy outcome, and pre-gestational diabetes	4	2	3
Naresh 2013 [4]	Live birth before 37 completed weeks of gestation	Exposed to any H1N1 influenza: 14.8 per 100 live births (21/142)<br><br>Exposed to severe H1N1 influenza: 25 per 100 live births (8/32)<br><br>Unexposed: 11.5 per 100 live births (82/710)	OR	Exposed to any H1N1 influenza: 1.33 (0.79–2.23)<br><br>Exposed to severe H1N1 influenza: 2.55 (1.11–5.87)	Exposed to any H1N1 influenza: 1.27 (0.75–2.15)<br><br>Exposed to severe H1N1 influenza: 2.07 (0.88–4.84)	Multivariable adjustment for study site, age, multiple gestation, and hypertensive disorders of pregnancy	4	2	3
Nieto-Pascual 2013 [5]	Birth before 37 completed weeks	Exposed: 4.0 per 100 births (3/75)	RR	0.40 (0.11–1.41) [d]	Not provided	No adjustment	3	0	3

	of gestation	Unexposed: 10.1 per 100 births (9/89)							
Martin 2013 [6]	Live birth with an ICD-9-CM diagnosis code indicating preterm delivery (644.2)	Respiratory illness present: 25.8 per 100 live birth delivery hospitalizations (4,420/17,136)<br><br>Respiratory illness absent: 7.3 per 100 live birth delivery hospitalizations (1,140,648/ 15,722,564)	OR	Not provided [g]	3.82 (3.53–4.14)	Multivariable adjustment for primary expected payer, hospital location, geographic location, and presence of high risk medical conditions (chronic cardiac disease, chronic pulmonary disease, diabetes mellitus, chronic renal disease, malignancies, immunosuppressive disorders)	3	2	2
Håberg 2013 [7]	Singleton live birth before 37 completed weeks of gestation	Estimates by influenza exposure were not provided.<br><br>Overall risk: 5.4 per 100 singleton live births (6,041 preterm births among 112,839 singleton live births)	HR	Not provided	Any exposure to pandemic time period: 1.00 (0.94–1.07)<br><br>Exposure to pandemic time period but no clinical influenza diagnosis: 1.00 (0.94–1.07)<br><br>Exposure to pandemic time period and clinical influenza diagnosis: 1.03 (0.84–1.25)	Multivariable adjustment for exposure to H1N1 vaccination, age, parity, marital status, use of nutritional supplements during pregnancy, smoking during pregnancy, history of earlier fetal death, and eight chronic medical conditions (asthma, hypertension, heart disease, kidney disease, rheumatoid arthritis, epilepsy, thyroid disease, or diabetes).	4	2	3
Hansen 2012 [8]	Live birth before 37 completed weeks of gestation	Exposed to seasonal influenza: 9.2 per 100 live births (35/381)<br><br>Exposed to H1N1 influenza: 11.0 per 100 live births (108/981)<br><br>Unexposed: 9.3 per 100 live births	OR	Exposed to seasonal influenza: 0.99 (0.70–1.40) [d]<br><br>Exposed to H1N1 influenza: 1.20 (0.99–1.47) [d]	Exposed to seasonal influenza: 0.82 (0.55–1.22)<br><br>Exposed to H1N1 influenza: 1.07 (0.82–1.40)	Multivariable adjustment for mother's age at delivery, plurality, race, study region, and smoking status	4	1	3

		(10,224/109,796)							
Morken 2011 [9]	Spontaneous onset of birth between 22+0 days and 36+6 days' gestation (among singleton live births)	Exposed week 0 to 13: 2.7 per 100 singleton live births (658/23,953)<br><br>Unexposed week 0 to 13: 2.8 per 100 singleton live births (1,212/43,357)<br><br>Exposed week 0 to 33: 2.5 per 100 singleton live births (822/33,305)<br><br>Unexposed week 0 to 33: 2.6 per 100 singleton live births (700/27,384)	HR	Not provided	Exposure before 17 weeks:<br>0.93 (0.83–1.03)<br><br>Exposure during gestational week 17 to 20:<br>0.89 (0.71–1.11)<br><br>Exposure during gestational week 21 to 30:<br>1.04 (0.92–1.18)	Multivariable adjustment for maternal age, parity, and smoking at the beginning of the pregnancy	3	1	3
Pierce 2011 [10]	Birth before 37 completed weeks of gestation<br><br>Computed per pregnancy (not per infant).	Exposed:<br>23.5 per 100 pregnancies (59/251)<br><br>Unexposed:<br>7.3 per 100 pregnancies (89/1,218)<br><br>National data:<br>7.9 per 100 pregnancies (36,283/459,758)	OR	Comparison with unexposed cohort:<br>3.9 (2.7–5.6)<br><br>Comparison with national data:<br>3.6 (2.7–4.8)	Comparison with unexposed cohort:<br>4.0 (2.7–5.9)<br><br>[After exclusion of iatrogenic preterm cesarean deliveries for maternal influenza, adjusted OR: 2.5 (1.6–3.9)]	Multivariable adjustment for socioeconomic status, ethnicity, parity, maternal age, smoking, multiple birth, and body mass index	4	1	3
McNeil 2011 [11]	Singleton, live birth before 37 completed weeks of gestation	Influenza-season respiratory-related hospitalization: 7.2 per 100 singleton live births (15/208)<br><br>No influenza-season respiratory-related hospitalization: 5.4 per 100 singleton live births (7,159/ 132,099)	OR	1.36 (0.80–2.30)	1.20 (0.71–2.04)	Multivariable adjustment for maternal high risk status, maternal age at birth, parity, maternal smoking during pregnancy, and infant sex	4	2	3

Rogers 2010 [12]	Birth before 37 completed weeks of gestation	Exposed: 4.9 per 100 births (4/81)<br><br>Unexposed: 5.2 per 100 births (1,622/30,983)	RR	0.94 (0.36–2.46) [d]	Not provided	No adjustment	4	0	2
Cox 2006 [13]	Live birth with an ICD-9-CM diagnosis code indicating preterm delivery (644)	Not provided	OR	Not provided [g]	4.08 (3.57–4.67)	Multivariable adjustment for maternal age, presence of high-risk conditions for which influenza vaccination is recommended, primary intended payer of care, hospital location, geographic region, and calendar year	3	2	2
Acs 2006 [14]	Live birth before 37 completed weeks of gestation	Exposed: 8.0 per 100 live births (147/1,838)<br><br>Unexposed: 9.2 per 100 live births (3,349/36,313)	POR	0.9 (0.7–1.0)	0.9 (0.8–1.1)	Multivariable adjustment for employment status, use of pregnancy supplements, and antifever or antimicrobial drugs	4	1	3
Hartert 2003 [15]	Birth before 37 completed weeks of gestation	Influenza-season respiratory-related hospitalization: 12.6 per 100 pregnancies (37/293)<br><br>No influenza-season respiratory-related hospitalization: 10.9 per 100 pregnancies (64/587)	OR	Not available due to matching	1.18 (0.77–1.82) [d]<br><br>Fully-adjusted OR not reported (p-value: 0.74)	Exposed and unexposed subjects were matched at a ratio of 1:2 on maternal age, race, gestational week of pregnancy on the date corresponding to the admission of the matched exposed woman, and presence or absence of high-risk conditions.<br><br>Additional multivariable adjustment for race, maternal smoking, marital status, level of education, and hospitalizations during the previous six months	3	1	3
Tuyishime 2003 [16]	Premature birth (not defined by authors) among live births (including singletons and multiples)	Exposed to ILI but no fever: 7.1 per 100 live births (16/224) [d]<br><br>Exposed to ILI with fever: 6.4 per 100	RR	ILI with no fever: 0.88 (0.45–1.73) [d]<br><br>ILI with fever: 0.80 (0.34–1.90) [d]	Not provided	No adjustment	4	0	3

		live births (7/108) [d]<br><br>Unexposed: 8.1 per 100 live births (15/185) [d]							
Irving 2000 [17] [f]	Preterm birth (not defined by authors)	--	--	--	--	Test positive (exposed) and test negative (unexposed) subjects were matched at a ratio of 1:1 on maternal age, gestational age of offspring, parity, and calendar month of delivery	--	--	--

HR, hazard ratio; RR, risk ratio; OR, odds ratio; POR, prevalence odds ratio; CI, confidence interval; ILI, influenza-like illness

[a] Risk of preterm birth per 100 among women classified as having (exposed) or not having (unexposed) influenza illness/infection during pregnancy.

[b] Selection (range 0 to 4), Comparability (range 0 to 2), Outcome/Exposure (range 0 to 3); maximum possible score = 9.

[c] Due to randomized assignment of influenza vaccination and the use of a placebo control group, we interpreted any differences in rates of preterm birth as attributable to differences in influenza disease between vaccinated and unvaccinated pregnant women. In this table, the influenza-exposed group refers to the placebo arm, and the influenza-unexposed group refers to the intervention arm.

[d] Estimated by review authors using raw data extracted from study.

[e] The RCT by Madhi et al.[1] was assessed using the Cochrane Collaboration tool for assessing risk of bias in randomized trials.[24] The risk of bias in the trial was rated as low.

[f] Odds ratios adjusted for maternal body mass index were additionally reported in the study, but have not been shown here since the results are not qualitatively different and 6% of records were excluded due to missing body mass index data.

[g] Not provided and cannot be computed without sampling weights.

[h] Although the authors reported the counts of preterm deliveries among test positive (n=0/181) and test negative women (n=3/180) in Table 2 of the original study, exposure groups were matched on gestational age at delivery in the design of the study, so preterm birth cannot be assessed.

Table S5. Publicly-reported baseline rates of pregnancy outcomes in four high-resource countries

Pregnancy outcome	United States [a]	Canada [b]	United Kingdom [c]	Norway [c]
Preterm birth (<37 weeks)	9.6% to 11.4%	7.7%	7.1%	6.3%
Low birth weight (<2,500 grams)	8.0%	8.1%	7.0%	4.9%
Fetal death per 1,000 births	6.1	6.7	5.1	3.7

[a] In 2013, preterm birth was 9.6% when using the obstetrical estimate of gestational age and 11.4% when using the last menstrual period-based estimate of gestational age. Source for preterm birth: Martin JA, Osterman MJK, Kirmeyer SE, Gregory ECW. Measuring gestational age in vital statistics data: Transitioning to the obstetric estimate. National vital statistics reports; vol 64 no 5. Hyattsville, MD: National Center for Health Statistics. 2015. [Data reported are for 2013]; Source for low birth weight: Martin JA, Hamilton BE, Osterman MJK, et al. Births: Final data for 2013. National vital statistics reports; vol 64 no 1. Hyattsville, MD: National Center for Health Statistics. 2015. [Data reported are for 2013]; Source for fetal death (≥20 weeks): MacDorman MF, Kirmeyer SE, Wilson EC. Fetal and perinatal mortality, United States, 2006. National vital statistics reports; vol 60 no 8. Hyattsville, MD: National Center for Health Statistics. 2012. Available at: http://www.cdc.gov/nchs/data/nvsr/nvsr60/nvsr60_08.pdf [Data reported are for 2006].

[b] Source for preterm birth and fetal death (≥20 weeks): Public Health Agency of Canada. Perinatal Health Indicators for Canada 2013: a Report of the Canadian Perinatal Surveillance System. Ottawa, 2013. [Data reported are for 2010]; Source for low birth weight: Statistics Canada Summary Tables for live births by birth weight and sex. Available at: http://www.statcan.gc.ca/tables-tableaux/sum-som/l01/cst01/health103b-eng.htm [Data reported are for 2011].

[c] Source: EURO-PERISTAT Project with SCPE and EUROCAT. European Perinatal Health Report. The health and care of pregnant women and babies in Europe in 2010.May 2013. Available at: www.europeristat.com [Data reported are for 2010].

Table S6. Assessment of heterogeneity among subgroups of studies reporting preterm birth <37 weeks

Subgroup	No. estimates [a]	Absolute risk of preterm birth <37 weeks (%) [b]		Pooled RR (95% CI) [c]	Heterogeneity [d]	
		Range among exposed women	Range among non-exposed women		Cochran's *Q*, df (*P*-value)	I^2 (95% CI)
**All studies (n=16)**	**17**	**4.0 to 25.8**	**5.2 to 11.5**	**1.34 (0.91–1.96)**	**667.7, 16 (<0.0001)**	**98% (97 to 98)**
**Newcastle-Ottawa Score (NOS)**						
0–5	0	---	---	---	---	---
6–7	8	4.0 to 25.8	5.2 to 10.9	1.40 (0.79–2.50)	362.3, 7 (<0.0001)	98% (98 to 98)
8–9 (highest quality) [e]	9	7.2 to 23.6	5.4 to 11.5	1.28 (0.95–1.73)	67.3, 8 (<0.0001)	88% (79 to 92)
**NOS ≥8, risk of diagnostic ascertainment bias not rated as 'very high', exposure not measured using self-reported questionnaire**						
Yes (highest quality) [e]	8	7.2 to 23.6	5.4 to 11.5	1.34 (0.91–1.96)	60.8, 7 (<0.0001)	89% (79 to 92)
No	9	4.0 to 25.8	5.2 to 10.9	1.31 (0.75–2.29)	473.5, 8 (<0.0001)	98% (98 to 99)
**Any adjustment for potential confounders (through matching, multivariable adjustment, randomization, or propensity scores)**						
Yes	13	7.2 to 25.8	5.4 to 11.5	1.51 (1.00–2.29)	651.6, 12 (<0.0001)	98% (98 to 98)
No	4	4.0 to 6.4	5.2 to 10.1	0.74 (0.42–1.28)	1.18, 3 (0.76)	0% (0 to 68)
**Risk of diagnostic ascertainment bias**						
Very high	2	25.8	7.3	3.89 (3.63–4.17)	0.67, 1 (0.41)	0% ([f])
High	8	4.3 to 23.6	5.2 to 10.9	1.39 (0.86–2.26)	57.1, 7 (<0.0001)	88% (78 to 92)
Medium	6	4.0 to 14.8	5.4 to 11.5	1.03 (0.94–1.13)	4.14, 5 (0.53)	0% (0 to 61)
Low	1	9.4	10.5	0.89 (0.68–1.17)	---	---
**Influenza season**						
2009 A (H1N1) pandemic	6	4.0 to 23.6	5.4 to 11.5	1.47 (0.91–2.39)	52.8, 5 (<0.0001)	91% (82 to 94)
Seasonal influenza	10	4.9 to 25.8	5.2 to 10.9	1.31 (0.79– 2.17)	553.2 (<0.0001)	98% (98 to 98)
2009 A (H1N1) pandemic and seasonal influenza	1	4.3	5.6	0.76 (0.10–5.94)	---	---
**Lab-confirmed influenza only**						
Yes	6	4.0 to 23.6	5.2 to 10.5	1.40 (0.75–2.61)	49.7, 5 (<0.0001)	90% (81 to 94)
No	11	4.3 to 25.8	5.4 to 10.9	1.31 (0.81–2.12)	602.0, 10 (<0.0001)	98% (98 to 99)
**Influenza illness severity**						
Severe influenza	7	4.9 to 25.8	5.2 to 10.9	2.44 (1.81–3.30)	60.4, 6 (<0.0001)	90% (82 to 94)
Mild-moderate influenza	10	4.0 to 14.8	5.4 to 11.5	0.99 (0.92–1.08)	6.52, 9 (0.83)	0% (0 to 53)
**Number of preterm births**						
<100	3	4.0 to 6.4	5.6 to 10.1	0.65 (0.33–1.28)	0.81, 2 (0.67)	0% (0 to 73)
100–499	4	12.6 to 23.5	7.3 to 11.5	1.52 (0.75–3.08)	39.3, 3 (<0.0001)	92% (83 to 95)
≥500	10	4.9 to 25.8	5.2 to 10.4	1.45 (0.90–2.36)	590.1, 9 (<0.0001)	99% (98 to 99)

**NOS ≥8, risk of diagnostic ascertainment bias not rated as 'very high', exposure not measured using self-reported questionnaire**						
**2009 A (H1N1) pandemic**	5	11.0 to 23.6 [g]	5.4 to 11.5	1.66 (1.01–2.74)	49.2, 4 (<0.0001)	92% (84 to 95)
Severe influenza	2	23.5 to 23.6	7.3 to 10.4	3.09 (1.86–5.11)	3.46, 1 (0.06)	71% ([e])
Mild-moderate influenza	3	11.0 to 14.8 [g]	5.4 to 11.5	1.06 (0.91–1.23)	0.54, 2 (0.76)	0% (0 to 73)
**Seasonal influenza**	3	7.2 to 9.4	5.4 to 10.5	0.91 (0.74–1.12)	1.39, 2 (0.50)	0% (0 to 73)
Severe influenza	1	7.2	5.4	1.20 (0.72–2.01)	--	--
Mild-moderate influenza	2	9.2 to 9.4	9.3 to 10.5	0.87 (0.69–1.09)	0.11, 1 (0.74)	0% ([e])

RR, risk ratio; CI, confidence interval; df, degrees of freedom

[a] There are 16 studies, but 17 estimates are shown since Hansen et al.[8] provided one estimate for the 2009 A (H1N1) pandemic and one for the 2008–2009 influenza season.

[b] The results from Morken et al.[9] were not reported in the range of preterm birth risks since only spontaneous preterm birth was studied, resulting in much lower risks.

[c] Adjusted ratio estimates were used when provided by the study, otherwise unadjusted ratio estimates were used.

[d] All heterogeneity estimates were computed using a random-effects model.

[e] The RCT by Madhi et al.[1] is listed with this category but was assessed using the Cochrane Collaboration tool for assessing risk of bias in randomized trials.[24] The risk of bias in the trial was rated as low.

[f] Not estimable with <2 degrees of freedom.

[g] Håberg et al.[7] did not provide the risk of preterm by exposure group. Overall risk in the study population was 5.4 preterm births per 100 singleton pregnancies.

Table S7. Results of studies reporting small-for-gestational-age (SGA) birth

First author and year of publication	Definition	Risk of SGA birth, by influenza infection/illness (n/N) [a]	Type of effect measure reported	Crude effect measure (95% CI)	Adjusted effect measure (95% CI)	Adjustment methods (i.e., matching, multivariable adjustment, propensity scores) and adjustment variables	Newcastle Ottawa Scale [b] Selection	Compar-ability	Outcome/ Exposure
Naresh 2013 [4]	Birth weight <10th percentile among all live births relative to a reference standard (singletons and multiples) [c]	Exposed to any H1N1 influenza: 8.5 per 100 live births (12/142)<br><br>Exposed to severe H1N1 influenza: 18.8 per 100 live births (6/32)<br><br>Unexposed: 7.4 per 100 live births (52/707)	OR	Exposed to any H1N1 influenza: 1.16 (0.60–2.24)<br><br>Exposed to severe H1N1 influenza: 2.19 (1.15–7.38)	Exposed to any H1N1 influenza: 1.14 (0.59–2.22)<br><br>Exposed to severe H1N1 influenza: 2.35 (1.03–5.36)	Multivariable adjustment for study site, age, multiple gestation, and hypertensive disorders of pregnancy	4	2	3
Nieto-Pascual 2013 [5]	Birth weight <10th percentile in the study population (singletons and multiples)	Exposed: 14.1 per 100 births (11/78)<br><br>Unexposed: 14.4 per 100 births (13/90)	RR	0.98 (0.46–2.05) [d]	Not provided	No adjustment	3	0	3
Hansen 2012 [8]	Live births below the 10th percentile of the sex-specific birth weight for gestational age distribution relative to a reference standard (published national birth weight percentiles)	Exposed to seasonal influenza: 12.0 per 100 live births [e]<br><br>Exposed to H1N1 influenza: 7.3 per 100 live births [e]<br><br>Unexposed: 7.2 per 100 live births [e]	OR	Not provided [e]	Exposed to seasonal influenza: 1.59 (1.15–2.20)<br><br>Exposed to H1N1 influenza: 0.99 (0.78–1.27)	Multivariable adjustment for mother's age at delivery, plurality, race, study region, and smoking status	4	1	3
McNeil 2011 [11]	Singleton live births below the 10th percentile of the sex-specific birth weight for gestational age distribution relative to a Canadian	Influenza-season respiratory-related hospitalization: 15.3 per 100 singleton live births (31/203)<br><br>No influenza-season respiratory-related	OR	1.68 (1.14–2.46)	1.66 (1.11–2.49)	Multivariable adjustment for maternal high risk status, maternal age at birth, parity, and maternal smoking during pregnancy	4	2	3

	reference standard [25]	hospitalization: 9.7 per 100 singleton live births (12,594/129,835)							
Irving 2000 [17]	Intrauterine growth restriction (not defined by authors)	Exposed: 2.8 per 100 births (5/181)<br><br>Unexposed: 3.9 per 100 births (7/180)	RR	Not available due to matching	0.71 (0.23–2.20)	Test positive (exposed) and test negative (unexposed) subjects were matched at a ratio of 1:1 on maternal age, gestational age of offspring, parity, and calendar month of delivery	4	1	3

CI, confidence interval; HR, hazard ratio; OR, odds ratio; RR, risk ratio; SGA, small for gestational age.

[a] Risk of SGA birth per 100 among women classified as having (exposed) or not having (unexposed) influenza illness/infection during pregnancy.

[b] Selection (range 0 to 4), Comparability (range 0 to 2), Outcome/Exposure (range 0 to 3); maximum possible score = 9.

[c] Hadlock's definition.

[d] Estimated by review authors using raw data extracted from study.

[e] 2,380 live births were missing information on birth weight; however, since the paper did not provide a breakdown by exposure category, the denominator values for exposure-specific risks were not available.

[f] Although not included in this table, Pierce et al., 2011[10] provided results for SGA in a rapid online response: birth weight <10th percentile (not sex-specific) was 11% in the infected cohort and 5% in the comparison cohort (p=0.051, OR: 1.56, 95% CI: 0.96–2.47).

Table S8. Results of studies reporting fetal death outcomes

First author and year of publication	Definition of fetal death	Risk of fetal death, by influenza infection/illness (n/N) [a]	Type of effect measure reported	Crude effect measure (95% CI)	Adjusted effect measure (95% CI)	Adjustment methods (i.e., matching, multivariable adjustment, propensity scores) and adjustment variables	Newcastle Ottawa Scale [b] Selection	Compar-ability	Outcome/ Exposure
Madhi 2014 [1]	(i) Miscarriage (20–27 weeks)<br><br>(ii) Stillbirth (≥28 weeks)	(i) Exposed: 4.8 per 1,000 births (5/1,037)[c]<br><br>Unexposed: 2.9 per 1,000 births (3/1,044)[c]<br><br>(ii) Exposed: 8.7 per 1,000 births (9/1,037)[c]<br><br>Unexposed: 14.4 per 1,000 births (15/1,044)[c]	RR	n/a due to randomization	(i) 1.68 (0.40–7.02) [d]<br><br>(ii) 0.60 (0.26–1.38) [d]	Randomization	--- [e]	--- [e]	--- [e]
Nieto-Pascual 2013 [5]	(i) Abortion [f]<br><br>(ii) Intrauterine fetal death [f]	(i) Exposed: 13.2 per 1,000 births (1/76)<br><br>Unexposed: 32.6 per 1,000 births (3/92)<br><br>(ii) Exposed: 13.3 per 1,000 births (1/75)<br><br>Unexposed: 11.2 per 1,000 births (1/89)	RR	(i) 0.40 (0.04–3.80) [d]<br><br>(ii) 1.19 (0.08–18.65) [d]	Not provided	No adjustment	3	0	3
Martin 2013 [6]	Intrauterine fetal demise or stillbirth (ICD–9–CM diagnosis codes 656.4, V27.1, V27.3, and V27.4) [g]	Respiratory illness present: 19.1 per 1,000 total delivery hospitalizations (333/17,468)<br><br>Respiratory illness absent: 6.5 per 1,000 total delivery hospitalizations (102,993/15,825,557)	OR	Not provided [h]	2.50 (1.97–3.18)	Multivariable adjustment for primary expected payer, hospital location, geographic location, and presence of high risk medical conditions (chronic cardiac disease, chronic pulmonary disease, diabetes mellitus, chronic renal disease, malignancies, immunosuppressive disorders)	3	2	2
Håberg 2013 [7]	Fetal death >12 weeks	Estimates by influenza exposure were not	HR	Any exposure to pandemic time	Any exposure to pandemic time	Multivariable adjustment for exposure to H1N1	4	2	3

	(spontaneous abortion or stillbirth)	provided.<br><br>Overall risk: 4.3 deaths per 1,000 births (492 fetal deaths among 113,331 eligible singleton pregnancies)		period: 1.15 (0.96–1.37)<br><br>Exposure to pandemic time period but no clinical influenza diagnosis:<br>1.11 (0.93–1.33)<br><br>Exposure to pandemic time period and clinical influenza diagnosis:<br>2.00 (1.20–3.32)	period: 1.26 (1.02–1.55)<br><br>Exposure to pandemic time period but no clinical influenza diagnosis:<br>1.23 (0.99–1.52)<br><br>Exposure to pandemic time period and clinical influenza diagnosis:<br>1.91 (1.07–3.41)	vaccination, maternal age, parity, marital status, use of nutritional supplements during pregnancy, smoking during pregnancy, history of previous fetal death, and eight chronic medical conditions (asthma, hypertension, heart disease, kidney disease, rheumatoid arthritis, epilepsy, thyroid disease, or diabetes).			
Pierce 2011 [10]	Fetal death ≥24 weeks	Exposed:<br>27.3 per 1,000 births (7/256)<br><br>Unexposed:<br>5.7 per 1,000 births (7/1,233)<br><br>National data:<br>5.1 per 1,000 births (4,043/799,047)	OR	Comparison with unexposed cohort:<br>4.9 (1.7–14.2)<br><br>Comparison with national data:<br>5.5 (2.6–11.7)	Comparison with unexposed cohort:<br>4.2 (1.4–12.4)	Multivariable adjustment for socioeconomic status, ethnicity, parity, maternal age, smoking, multiple birth, and body mass index	4	1	3
Hartert 2003 [15]	Fetal death identified by fetal death registrations [f]	Influenza-season respiratory-related hospitalization: 10.2 per 1,000 pregnancies (3/293)<br><br>No influenza-season respiratory-related hospitalization: 3.4 per 1,000 pregnancies (2/587)	OR	Not available due to matching	3.03 (0.50–18.2) [d]<br><br>Fully-adjusted OR not reported (p-value: 0.34)	Exposed and unexposed subjects were matched at a ratio of 1:2 on maternal age, race, gestational week of pregnancy on the date corresponding to the admission of the matched exposed woman, and presence or absence of high-risk conditions.<br><br>Additional multivariable adjustment for race, maternal smoking, marital status, level of education, and hospitalizations during the previous six months	3	1	3
Irving 2000 [17]	Fetal death [f]	Exposed: 1/180	Not reported	Not reported	Not reported	Test positive (exposed) and test negative (unexposed)	4	0	3

		Unexposed: 0/180				subjects were matched at a ratio of 1:1 on maternal age, gestational age of offspring, parity, and calendar month of delivery			
Stanwell-Smith 1994 [18]	Spontaneous abortion or stillbirth [f]	(i) Cases: 10/11 had symptoms of ILI<br><br>Controls: 4/9 had symptoms of ILI<br><br>(ii) Cases: 8/8 were seropositive for influenza A<br><br>Controls: 0/6 were seropositive for influenza A	OR	(i) 12.5 (1.09–143) [d]<br><br>(ii) Not estimable	Not provided	No adjustment	4	0	3
Korones 1970 [20]	Stillbirth [f]	Exposed: 19.6 per 1,000 (1/51)<br><br>Unexposed: 19.6 per 1,000 (1/51)	RR	Not available due to matching	1.00 (0.06–15.56) [d]	Exposed and unexposed subjects were matched at a ratio of 1:1 on race, maternal age, date of blood collection, length of gestation, sex of offspring, and collaborating institution	4	1	3
Wilson 1969 [21]	Fetal death [f]	Exposed: 30.1 per 1,000 births (9/299)<br><br>Unexposed: 13.3 per 1,000 (2.5/188) [i]	RR	2.26 (0.56–9.08)	Not provided	No adjustment	4	0	3

HR, hazard ratio; RR, risk ratio; OR, odds ratio; CI, confidence interval; ICD–9–CM, International Classification of Diseases, Ninth Revision–Clinical Modification; ILI, influenza-like illness

[a] Risk of fetal death among women classified as having (exposed) or not having (unexposed) influenza illness/infection during pregnancy.

[b] Selection (range 0 to 4), Comparability (range 0 to 2), Outcome/Exposure (range 0 to 3); maximum possible score = 9.

[c] Due to randomized assignment of influenza vaccination and the use of a placebo control group, we interpreted any differences in rates of preterm birth as attributable to differences in influenza disease between vaccinated and unvaccinated pregnant women. In this table, the influenza-exposed group refers to the placebo arm, and the influenza-unexposed group refers to the intervention arm.

[d] Estimated by review authors using raw data extracted from study.

[e] The RCT by Madhi et al.[1] was assessed using the Cochrane Collaboration tool for assessing risk of bias in randomized trials.[24] The risk of bias in the trial was rated as low.

[f] Authors did not provide the gestational age range/threshold underlying their definition.

[g] ICD–9–CM diagnosis code definitions: 656.4=intrauterine death affecting management of mother; V27.1=single stillborn (outcome of delivery); V27.3=twins, one live born and one stillborn (outcome of delivery); V27.4=twins, both stillborn (outcome of delivery).

[h] Not provided and cannot be computed without sampling weights.

[i] 0.5 indicates a twin sibling

Table S9. Results of studies reporting preterm birth <32 weeks

First author and year of publication	Definition	Risk of preterm birth <32 weeks, by influenza infection/illness (n/N) [a]	Type of effect measure reported	Crude effect measure (95% CI)	Adjusted effect measure (95% CI)	Adjustment methods (i.e., matching, multivariable adjustment, propensity scores) and adjustment variables	Newcastle Ottawa Scale [b] Selection	Compar-ability	Outcome/ Exposure
Doyle 2013 [3]	Live birth before 32 completed weeks of gestation	Exposed: 6.8 per 100 live births (13/191)<br><br>Unexposed: 1.7 per 100 live births (5,152/299,885)	OR	4.18 (2.38–7.34)	3.04 (1.57–5.90) [c]	Multivariable adjustment for maternal age, maternal race, maternal ethnicity, maternal education, maternal marital status, plurality, infant sex, tobacco use during pregnancy, alcohol use during pregnancy, previous preterm delivery, previous poor pregnancy outcome, and pre-gestational diabetes	4	2	3
Pierce 2011 [10]	Birth before 32 completed weeks of gestation<br><br>Computed per pregnancy (not per infant).	Exposed: 7.2 per 100 pregnancies (18/251)<br><br>Unexposed: 1.5 per 100 pregnancies (18/1,218)<br><br>National data: 2.4 per 100 pregnancies (10,932/460,033)	OR	Comparison with unexposed cohort: 5.2 (2.6–10.0)<br><br>Comparison with national data: 3.2 (2.0–5.1)	Comparison with unexposed cohort: 4.9 (2.4–10.0)	Multivariable adjustment for socioeconomic status, ethnicity, parity, maternal age, smoking, multiple birth, and body mass index	4	1	3

OR, odds ratio; CI, confidence interval

[a] Risk of preterm birth <32 weeks among women classified as having (exposed) or not having (unexposed) influenza illness/infection during pregnancy.

[b] Selection (range 0 to 4), Comparability (range 0 to 2), Outcome/Exposure (range 0 to 3); maximum possible score = 9.

[c] Odds ratios adjusted for maternal body mass index were additionally reported in the study, but have not been shown here as the results are not qualitatively different and 6% of records were excluded due to missing body mass index data.

Table S10. Results of studies reporting preterm birth using other gestational age thresholds

First author and year of publication	Definition	Risk of preterm birth using other gestational age thresholds, by influenza infection/illness (n/N) [a]	Type of effect measure reported	Crude effect measure (95% CI)	Adjusted effect measure (95% CI)	Adjustment methods (i.e., matching, multivariable adjustment, propensity scores) and adjustment variables	Newcastle Ottawa Scale [b]		
							Selection	Compar-ability	Outcome/ Exposure
Doyle 2013 [3]	Live birth before 28 completed weeks of gestation	Exposed: 4.2 per 100 live births (8/191)<br><br>Unexposed: 0.7 per 100 live births (2,258/299,885)	OR	5.77 (2.84–11.71)	4.19 (1.82–9.63) [c]	Multivariable adjustment for maternal age, maternal race, maternal ethnicity, maternal education, maternal marital status, plurality, infant sex, tobacco use during pregnancy, alcohol use during pregnancy, previous preterm delivery, previous poor pregnancy outcome, and pre-gestational diabetes	4	2	3
Rogers 2010 [12]	Birth before 34 completed weeks of gestation	Exposed:<br>1.2 per 100 births (1/81)<br><br>Unexposed:<br>2.3 per 100 births (703/30,983)	RR	0.54 (0.08–3.82) [d]	Not provided	No adjustment	4	0	2

HR, hazard ratio; RR, risk ratio; OR, odds ratio; CI, confidence interval

[a] Risk of preterm birth among women classified as having (exposed) or not having (unexposed) influenza illness/infection during pregnancy.

[b] Selection (range 0 to 4), Comparability (range 0 to 2), Outcome/Exposure (range 0 to 3); maximum possible score = 9.

[c] Odds ratios adjusted for maternal body mass index were additionally reported in the study, but have not been shown here as the results are not qualitatively different and 6% of records were excluded due to missing body mass index data.

[d] Estimated by review authors using raw data extracted from study.

Table S11. Results of studies reporting mean gestational age

First author and year of publication	Definition	Mean gestational age, by influenza infection/illness (95% CI or SD) [a]	Type of effect measure reported	Crude effect measure (95% CI)	Adjusted effect measure (95% CI)	Adjustment methods (i.e., matching, multivariable adjustment, propensity scores) and adjustment variables	Newcastle Ottawa Scale [b]		
							Selection	Compar-ability	Outcome/ Exposure
Doyle 2013 [3]	Mean gestational age among live births	Exposed: 37.3 weeks<br><br>Unexposed: 38.3 weeks	Mean difference	1 week [c] (p-value<0.001)	Not provided	No adjustment	4	0	3
Naresh 2013 [4]	Mean gestational age among live births (including singletons and multiples)	Exposed to any H1N1 influenza: 38.6 weeks (38.2–38.9)<br><br>Exposed to severe H1N1 influenza: 38.1 weeks (37.4–38.7)<br><br>Unexposed: 38.7 weeks (38.6–38.9)	Mean difference	Exposed to any H1N1 influenza: -0.1 weeks [c] (p-value: 0.39)<br><br>Exposed to severe H1N1 influenza: -0.6 weeks [c] (p-value: 0.10)	Exposed to any H1N1 influenza: -0.11 weeks (p-value: 0.59)<br><br>Exposed to severe H1N1 influenza: -0.51 weeks (p-value: 0.26)	Multivariable adjustment for study site, age, multiple gestation, and hypertensive disorders of pregnancy	4	2	3
Nieto-Pascual 2013 [5]	Mean gestational age among total births (including singletons and multiples)	Exposed: 39.4 weeks (SD: 1.3)<br><br>Unexposed: 39.1 weeks (SD: 1.8)	Mean difference	0.3 weeks [c] (p-value: 0.35)	Not provided	No adjustment	2	0	3
Rogers 2010 [12]	Mean gestational age among total births (including singletons and multiples)	Exposed: 38.8 weeks (SD: 2.9)<br><br>Unexposed: 38.5 weeks (SD: 4.9)	Mean difference	0.3 weeks [c] (p-value: 0.26)	Not provided	No adjustment	4	0	2
Acs 2006 [14]	Mean gestational age among live births (including singletons and multiples)	Exposed: 39.5 weeks (SD: 1.9)<br><br>Unexposed: 39.4 weeks (SD: 2.1)	Mean difference	0.1 weeks [c] (p-value: 0.02)	Not provided (p-value: 0.12)	Multivariable adjustment for employment status, use of pregnancy supplements, and antifever or antimicrobial drugs	4	1	3
Tuyishime 2003 [16]	Mean gestational age among live births (including singletons and multiples)	Exposed to ILI but no fever: 38.6 weeks<br><br>Exposed to ILI with fever: 38.6 weeks<br><br>Unexposed: 38.7	Mean difference	ILI with no fever: -0.1 weeks [c] (p-value: 0.89)<br><br>ILI with fever: -0.1 weeks [c]	Not provided	No adjustment	4	0	3

weeks

CI, confidence interval; SD, standard deviation
[a] Mean gestational age among women classified as having (exposed) or not having (unexposed) influenza illness/infection during pregnancy.
[b] Selection (range 0 to 4), Comparability (range 0 to 2), Outcome/Exposure (range 0 to 3); maximum possible score = 9.
[c] Estimated by review authors using raw data extracted from study.

Table S12. Results of studies reporting low birth weight (<2,500 grams), by method of accounting for gestational age

First author and year of publication	Definition	Risk of birth weight <2,500 grams, by influenza infection/illness (n/N) [a]	Type of effect measure reported	Crude effect measure (95% CI)	Adjusted effect measure (95% CI)	Adjustment methods (i.e., matching, multivariable adjustment, propensity scores) and adjustment variables	Newcastle Ottawa Scale [b] Selection	Compar-ability	Outcome/ Exposure
**Restriction to term gestation or matching on gestational age**									
Håberg 2013 [7]	Birth weight below 2,500 grams among singleton live births delivered at or after 37 weeks of gestation (term low birth weight)	Overall risk: 3.4 per 100 singleton, term live births (3,866 among 112,092 singleton live births ≥37 weeks)<br><br>Comparable estimates by influenza exposure were not provided.	HR	Not reported	Any exposure to pandemic time period:<br>1.08 (0.94–1.24)<br><br>Exposure to pandemic time period but no clinical influenza diagnosis:<br>1.07 (0.93–1.24)<br><br>Exposure to pandemic time period and clinical influenza diagnosis:<br>1.28 (0.84–1.95)	Multivariable adjustment for exposure to exposure to H1N1 vaccination, age, parity, marital status, use of nutritional supplements during pregnancy, smoking during pregnancy, history of earlier fetal death, and eight chronic medical conditions (asthma, hypertension, heart disease, kidney disease, rheumatoid arthritis, epilepsy, thyroid disease, or diabetes).	4	2	3
Hartert 2003 [15]	Birth weight below 2,500 grams among all births	Influenza-season respiratory-related hospitalization: 10.6 per 100 pregnancies (31/293)<br><br>No influenza-season respiratory-related hospitalization: 8.2 per 100 pregnancies (48/587)	OR	Not available due to matching	1.33 (0.83–2.14) [c]<br><br>Fully adjusted OR not reported (p-value 0.68)	Exposed and unexposed subjects were matched at a ratio of 1:2 on maternal age, race, gestational week of pregnancy on the date corresponding to the admission of the matched exposed woman, and presence or absence of high-risk conditions.<br><br>Additional multivariable adjustment for race, maternal smoking, marital status, level of education, and hospitalizations during the previous six months	3	1	3

**Multivariable adjustment for gestational age**									
Hansen 2012 [8]	Birth weight below 2,500 grams among live births	Exposed to seasonal influenza: 7.0 per 100 live births [d]<br><br>Exposed to A (pH1N1) influenza: 7.3 per 100 live births [d]<br><br>Unexposed: 6.5 per 100 live births)	OR	Not provided [d]	Exposed to seasonal influenza: 1.11 (0.59–2.07)<br><br>Exposed to A (pH1N1) influenza: 1.14 (0.80–1.64)	Multivariable adjustment for mother's age at delivery, plurality, race, study region, smoking status, and gestational age at birth	4	1	3
McNeil 2011 [11]	Birth weight below 2,500 grams among singleton live births	Influenza-season respiratory-related hospitalization: 6.3 per 100 singleton live births (13/208)<br><br>No influenza-season respiratory-related hospitalization: 4.6 per 100 singleton live births (6,035/ 132,099)	OR	1.39 (0.80–2.45)	1.30 (0.59–2.86)	Multivariable adjustment for maternal high risk status, maternal age at birth, parity, maternal smoking during pregnancy, infant sex, and gestational age at birth	4	2	3
**No restriction or adjustment for gestational age**									
Madhi 2014 [1]	Birth weight below 2,500 grams among live births	Exposed: 11.9 per 100 live births (122/1,021) [e]<br><br>Unexposed: 13.0 per 100 live births (133/1,024) [e]	RR	n/a due to randomization	0.92, 0.72–1.18 [c]	Randomization	--- [f]	--- [f]	--- [f]
Doyle 2013 [3]	Birth weight below 2,500 grams among live births	Exposed: 15.7 per 100 live births (30/191)<br><br>Unexposed: 8.7 per 100 live births (25,998/300,398)	OR	1.97 (1.33–2.91)	1.67 (1.06–2.62) [g]	Multivariable adjustment for maternal age, maternal race, maternal ethnicity, maternal education, maternal marital status, plurality, infant sex, tobacco use during pregnancy, alcohol use during pregnancy, previous preterm delivery, previous poor pregnancy outcome, and pre-gestational diabetes	4	2	3
Pierce 2011 [10]	Birth weight below 2,500 grams among	Exposed:<br>19.5 per 100 births	OR	Comparison with unexposed cohort:	Comparison with unexposed cohort:	Multivariable adjustment for socioeconomic status,	4	1	3

	total births (including singletons and multiples)	(50/256)<br><br>Unexposed:<br>7.6 per 100 births (94/1,231)<br><br>National data:<br>7.4 per 100 births (57,072/770,273)		2.9 (2.0–4.3)<br><br>Comparison with national data:<br>3.0 (2.2–4.1)	3.2 (2.1–4.9)	ethnicity, parity, maternal age, smoking, multiple birth, and body mass index			
Rogers 2010 [12]	Birth weight below 2,500 grams among total births (including singletons and multiples)	Exposed: 8.6 per 100 births (7/81)<br><br>Unexposed: 5.9 per 100 births (1,814/30,983)	RR	1.48 (0.73–3.00)	Not provided	No adjustment	4	0	2
Acs 2006 [14]	Birth weight below 2,500 grams among live births	Exposed: 4.7 per 100 live births (87/1,838)<br><br>Unexposed: 5.7 per 100 live births (2,080/36,313)	POR	0.9 (0.7–1.0)	0.9 (0.7–1.1)	Multivariable adjustment for employment status, use of pregnancy supplements, and antifever – antimicrobial drugs	4	1	3
Tuyishime 2003 [16]	Low birth weight (not defined by authors) among live births (including singletons and multiples)	Exposed to ILI but no fever: 5.8 per 100 live births (13/224) [c]<br><br>Exposed to ILI with fever: 3.7 per 100 live births (4/108) [c]<br><br>Unexposed: 9.1 per 100 live births (17/185) [c]	RR	ILI with no fever: 0.63 (0.32–1.27) [c]<br><br>ILI with fever: 0.40 (0.14–1.17) [c]	Not provided	No adjustment	4	0	3
Wilson 1969 [21]	Birth weight below 2,500 grams among live births	Exposed: 7.5 per 100 live births (22.5/299)<br><br>Unexposed: 6.4 per 100 live births (12/188)	RR	1.18 (0.60–2.32) [c]	Not provided	No adjustment	3	0	3

HR, hazard ratio; RR, risk ratio; OR, odds ratio; CI, confidence interval

[a] Risk of low birth weight among women classified as having (exposed) or not having (unexposed) influenza illness/infection during pregnancy.

[b] Selection (range 0 to 4), Comparability (range 0 to 2), Outcome/Exposure (range 0 to 3); maximum possible score = 9.

[c] Estimated by review authors using raw data extracted from study.

[d] 2,380 live births were missing information on birth weight; however, since the paper did not provide a breakdown by exposure category, the denominator values for exposure-specific risks were not available.
[e] Due to randomized assignment of influenza vaccination and the use of a placebo control group, we interpreted any differences in rates of low birth weight as attributable to differences in influenza disease between vaccinated and unvaccinated pregnant women. In this table, the influenza-exposed group refers to the placebo arm, and the influenza-unexposed group refers to the intervention arm.
[f] The RCT by Madhi et al.[1] was assessed using the Cochrane Collaboration tool for assessing risk of bias in randomized trials.[24] The risk of bias in the trial was rated as low.
[g] In the text, Doyle et al.[3] note that the OR for low birth weight was not statistically significant after adjusting for gestational age in addition to the other variables listed in the table; however, no point estimates for this additional adjustment were provided.

Table S13. Results of studies reporting mean birth weight, by method of accounting for gestational age

First author and year of publication	Definition	Mean birth weight, by influenza infection/illness (95% CI or SD) [a]	Type of effect measure reported	Crude effect measure (95% CI)	Adjusted effect measure (95% CI)	Adjustment methods (i.e., matching, multivariable adjustment, propensity scores) and adjustment variables	Newcastle Ottawa Scale [b] Selection	Compar-ability	Outcome/ Exposure
**Restriction to term gestation or matching on gestational age**									
McNeil 2011 [11]	(i) Mean birth weight among all singleton live births<br><br>(ii) Mean birth weight among singleton live births delivered at or after 37 weeks of gestation (term infants)	(i) Influenza-season respiratory-related hospitalization: 3,360 grams (SD: 635)<br><br>No influenza-season respiratory-related hospitalization: 3,468 grams (SD: 584)<br><br>(ii) Influenza-season respiratory-related hospitalization: 3,449 grams (SD: 498)<br><br>No influenza-season respiratory-related hospitalization: 3,531 grams (SD: 504)	Mean difference	(i) -108 grams [c]<br><br>(ii) -83 grams [c]	(i) -86 grams [c]<br><br>(ii) -87 grams [c]	Multivariable adjustment for maternal high risk status, maternal age at birth, parity, maternal smoking during pregnancy, infant sex, and gestational age at birth	4	2	3
Acs 2006 [14]	(i) Mean birth weight among live births (including singletons and multiples)<br><br>(ii) Mean birth weight among live births 37–41 weeks (including singletons and multiples)	(i) Exposed: 3,311 grams (SD: 492)<br><br>Unexposed: 3,274 grams (SD: 512)<br><br>(ii) Exposed: 3,347 grams (SD: 435)<br><br>Unexposed: 3,322 grams (SD: 430)	Mean difference	(i) 37 grams [d] (p-value: 0.002)<br><br>(ii) 25 grams [d]	Not provided (p-value: 0.08)	Multivariable adjustment for employment status, use of pregnancy supplements, and antifever or antimicrobial drugs	4	1	3
Hartert 2003 [15]	Mean birth weight among singleton	Influenza-season respiratory-related	Mean difference	Not available due to matching	-99 grams [d]	Exposed and unexposed subjects were matched at a	3	1	3

	live births	hospitalization: 3,124 grams (SD: 614)<br><br>No influenza-season respiratory-related hospitalization: 3,223 grams (SD: 609)			Fully-adjusted mean difference not provided (p-value: 0.28)	ratio of 1:2 on maternal age, race, gestational week of pregnancy on the date corresponding to the admission of the matched exposed woman, and presence or absence of high-risk conditions.<br><br>Additional multivariable adjustment for race, maternal smoking, marital status, level of education, and hospitalizations during the previous six months			
Irving 2000 [17]	Mean birth weight	Exposed: 3.40 kg (SD: 0.54)<br><br>Unexposed: 3.33 kg (SD: 0.58)	Not reported	[e]	[e]	Test positive (exposed) and test negative (unexposed) subjects were matched at a ratio of 1:1 on maternal age, <u>gestational age of offspring</u>, parity, and calendar month of delivery	4	1	3
**No restriction or adjustment for gestational age**									
Madhi 2014 [1]	Median birth weight among live births	Exposed: 3.1 kg (range: 0.7–4.8) [f]<br><br>Unexposed: 3.1 kg (range: 0.5–4.6) [f]	Not reported	n/a due to randomization	[e]	Randomization	--- [g]	--- [g]	--- [g]
Doyle 2013 [3]	Mean birth weight among live births	Exposed: 3,030 grams<br><br>Unexposed: 3,229 grams	Mean difference	-199 grams (p-value<0.001)	Not provided	No adjustment	4	0	3
Naresh 2013 [4]	Mean birth weight among live births (including singletons and multiples)	Exposed to any A (pH1N1) influenza: 3,208.6 grams (3,109.2–3,308.0)<br><br>Exposed to severe A (pH1N1)influenza: 3,013.0 grams (2,785.8–3,240.2)<br><br>Unexposed: 3,223.3 grams (3,174.0–3,272.5)	Mean difference	Exposed to any A (pH1N1)influenza: -14.7 grams [d] (p-value: 0.81)<br><br>Exposed to severe A (pH1N1)influenza: -210.3 grams [d] (p-value: 0.08)	Exposed to any A (pH1N1)influenza: 6.91 grams (p-value: 0.76)<br><br>Exposed to severe A (pH1N1) influenza: -222.1 grams (p-value: 0.08)	Multivariable adjustment for study site, age, multiple gestation, and hypertensive disorders of pregnancy	4	2	3
Nieto-Pascual	Mean birth weight	Exposed: 3,233.7	Mean difference	27.6 grams [d] (p-	Not provided	No adjustment	3	0	3

2013 [5]	(including singletons and multiples)	grams (SD: 504)<br><br>Unexposed: 3,206.1 grams (SD: 533)		value: 0.732)					
Pierce 2011 [10]	Mean birth weight among total births (including singletons and multiples)	Exposed: 3,073 grams (SD: 774)<br><br>Unexposed: 3,342 grams (SD: 614)	Mean difference	Comparison with unexposed cohort: -270 grams (-356 to -183)	Comparison with unexposed cohort: -255 (-353 to -156)	Multivariable adjustment for socioeconomic status, ethnicity, parity, maternal age, smoking, multiple birth, and body mass index	4	1	3
Rogers 2010 [12]	Mean birth weight among total births (including singletons and multiples)	Exposed: 3,241 grams (SD: 542)<br><br>Unexposed: 3,322 grams (SD: 583)	Mean difference	-81 grams [d] (p-value: 0.22)	Not provided	No adjustment	4	0	2
Tuyishime 2003 [16]	Mean birth weight among live births (including singletons and multiples)	Exposed to ILI but no fever: 3,350 grams<br><br>Exposed to ILI with fever: 3,384 grams<br><br>Unexposed: 3,267 grams	Mean difference	ILI with no fever: 83 grams [d] (p-value: 0.17)<br><br>ILI with fever: 117 grams [d]	Not provided	No adjustment	4	0	3
Griffiths 1980 [19]	Mean birth weight among singleton live births	Exposed: 3.30 kg<br><br>Unexposed: 3.30 kg	Not reported	[e]	[e]	No adjustment	4	0	3
Korones 1970 [20]	Mean birth weight	Exposed: 3.2 kg<br><br>Unexposed: 3.1 kg	Not reported	[e]	[e]	Exposed and unexposed subjects were matched at a ratio of 1:1 on race, maternal age, date of blood collection, length of gestation, sex of offspring, and collaborating institution	4	1	3

CI, confidence interval; SD, standard deviation

[a] Mean birth weight among women classified as having (exposed) or not having (unexposed) influenza illness/infection during pregnancy.

[b] Selection (range 0 to 4), Comparability (range 0 to 2), Outcome/Exposure (range 0 to 3); maximum possible score = 9.

[c] Measure of variance not provided; all P-values <0.05.

[d] Estimated by review authors using raw data extracted from study.

[e] Insufficient information to compute a mean difference in grams.

[f] Due to randomized assignment of influenza vaccination and the use of a placebo control group, we interpreted any differences in birth weight as attributable to differences in influenza disease between vaccinated and unvaccinated pregnant women. In this table, the influenza-exposed group refers to the placebo arm, and the influenza-unexposed group refers to the intervention arm.

[g] The RCT by Madhi et al.[1] was assessed using the Cochrane Collaboration tool for assessing risk of bias in randomized trials.[24] The risk of bias in the trial was rated as low.

References

1. Madhi S a., Cutland CL, Kuwanda L, et al. Influenza Vaccination of Pregnant Women and Protection of Their Infants. *N Engl J Med*. 2014;371(10):918-931. doi:10.1056/NEJMoa1401480.

2. Ahrens K a, Louik C, Kerr S, Mitchell A a, Werler MM. Seasonal influenza vaccination during pregnancy and the risks of preterm delivery and small for gestational age birth. *Paediatr Perinat Epidemiol*. 2014;28(6):498-509. doi:10.1111/ppe.12152.

3. Doyle TJ, Goodin K, Hamilton JJ. Maternal and neonatal outcomes among pregnant women with 2009 pandemic influenza A(H1N1) illness in Florida, 2009-2010: a population-based cohort study. *PLoS One*. 2013;8(10):e79040. doi:10.1371/journal.pone.0079040.

4. Naresh a, Fisher BM, Hoppe KK, et al. A multicenter cohort study of pregnancy outcomes among women with laboratory-confirmed H1N1 influenza. *J Perinatol*. 2013;33(12):939-943. doi:10.1038/jp.2013.110.

5. Nieto-Pascual L, Arjona-Berral JE, Marín-Martín EM, Muñoz-Gomariz E, Ilich I, Castelo-Branco C. Early prophylactic treatment in pregnant women during the 2009-2010 H1N1 pandemic: obstetric and neonatal outcomes. *J Obstet Gynaecol*. 2013;33(2):128-134. doi:10.3109/01443615.2012.740526.

6. Martin A, Cox S, Jamieson DJ, Whiteman MK, Kulkarni A, Tepper NK. Respiratory illness hospitalizations among pregnant women during influenza season, 1998-2008. *Matern Child Health J*. 2013;17(7):1325-1331. doi:10.1007/s10995-012-1135-3.

7. Håberg SE, Trogstad L, Gunnes N, et al. Risk of fetal death after pandemic influenza virus infection or vaccination. *N Engl J Med*. 2013;368(4):333-340. doi:10.1056/NEJMoa1207210.

8. Hansen C, Desai S, Bredfeldt C, et al. A large, population-based study of 2009 pandemic Influenza A virus subtype H1N1 infection diagnosis during pregnancy and outcomes for mothers and neonates. *J Infect Dis*. 2012;206(8):1260-1268. doi:10.1093/infdis/jis488.

9. Morken N-H, Gunnes N, Magnus P, Jacobsson B. Risk of spontaneous preterm delivery in a low-risk population: the impact of maternal febrile episodes, urinary tract infection, pneumonia and ear-nose-throat infections. *Eur J Obstet Gynecol Reprod Biol*. 2011;159(2):310-314. doi:10.1016/j.ejogrb.2011.08.006.

10. Pierce M, Kurinczuk JJ, Spark P, Brocklehurst P, Knight M. Perinatal outcomes after maternal 2009/H1N1 infection: national cohort study. *BMJ*. 2011;342(jun14 2):d3214. doi:10.1136/bmj.d3214.

11. McNeil S a, Dodds L a, Fell DB, et al. Effect of respiratory hospitalization during pregnancy on infant outcomes. *Am J Obstet Gynecol*. 2011;204(6 Suppl 1):S54-S57. doi:10.1016/j.ajog.2011.04.031.

12. Rogers VL, Sheffield JS, Roberts SW, et al. Presentation of seasonal influenza A in pregnancy: 2003-2004 influenza season. *Obstet Gynecol*. 2010;115(5):924-929. doi:10.1097/AOG.0b013e3181da0c5e.

13. Cox S, Posner SF, McPheeters M, Jamieson DJ, Kourtis AP, Meikle S. Hospitalizations with respiratory illness among pregnant women during influenza season. *Obstet Gynecol*. 2006;107(6):1315-1322. doi:10.1097/01.AOG.0000218702.92005.bb.

14. Acs N, Bánhidy F, Puhó E, Czeizel AE. Pregnancy complications and delivery outcomes of pregnant women with influenza. *J Matern Fetal Neonatal Med*. 2006;19(3):135-140. doi:10.1080/14767050500381180.

15. Hartert T V, Neuzil KM, Shintani AK, et al. Maternal morbidity and perinatal outcomes among pregnant women with respiratory hospitalizations during influenza season. *Am J Obstet Gynecol*. 2003;189(6):1705-1712. doi:10.1016/S0002-9378(03)00857-3.

16. Tuyishime J-D, De Wals P, Moutquin J-M, Frost E. Influenza-like illness during pregnancy: results from a study in the eastern townships, Province of Quebec. *J Obstet Gynaecol Can*. 2003;25(12):1020-1025.

17. Irving WL, James DK, Stephenson T, et al. Influenza virus infection in the second and third trimesters of pregnancy: a clinical and seroepidemiological study. *BJOG*. 2000;107(10):1282-1289.

18. Stanwell-Smith R, Parker AM, Chakraverty P, Soltanpoor N, Simpson CN. Possible association of influenza A with fetal loss: investigation of a cluster of spontaneous abortions and stillbirths. *Commun Dis Rep CDR Rev*. 1994;4(3):R28-R32.

19. Griffiths PD, Ronalds CJ, Heath RB. A prospective study of influenza infections during pregnancy. *J Epidemiol Community Health*. 1980;34(2):124-128.

20. Korones SB, Todaro J, Roane JA, Sever JL. Maternal virus infection after the first trimester of pregnancy and status of offspring to 4 years of age in a predominantly Negro population. *J Pediatr*. 1970;77(2):245-251.

21. Wilson MG, Stein AM. Teratogenic effects of asian influenza. A n extended study. *JAMA*. 1969;210(2):336-337.

22. Yates L, Pierce M, Stephens S, et al. Influenza A/H1N1v in pregnancy: an investigation of the characteristics and management of affected women and the relationship to pregnancy outcomes for mother and infant. *Health Technol Assess*. 2010;14(34):109-182. doi:10.3310/hta14340-02.

23. Dodds L, McNeil SA, Fell DB, et al. Impact of influenza exposure on rates of hospital admissions and physician visits because of respiratory illness among pregnant women. *CMAJ*. 2007;176(4):463-468. doi:10.1503/cmaj.061435.

24. Higgins JPT, Altman DG, Gøtzsche PC, et al. The Cochrane Collaboration's tool for assessing risk of bias in randomised trials. *BMJ*. 2011;343:d5928.

25. Kramer MS, Platt RW, Wen SW, et al. A new and improved population-based Canadian reference for birth weight for gestational age. *Pediatrics*. 2001;108(2):E35.
