# Supplementary material for: Maternal influenza and birth outcomes: systematic review of comparative studies
Source: BJOG. 2016 Jun 6;124(1):48–59. doi: 10.1111/1471-0528.14143 (PMC5216449; doi:10.1111/1471-0528.14143)
Supplement: Supplementary file 3 — Appendix S1. Full search strategy with results [file BJO-124-48-s003.pdf]

Appendix S1. Final search strategies with results (as of December 5, 2014)

Database: Embase Classic+Embase <1947 to 2014 December 04>, Ovid MEDLINE(R) In-Process & Other Non-Indexed Citations and Ovid MEDLINE(R) <1946 to Present> Search Strategy:

----

1 Influenza, Human/ (91779)
2 (influenza or flu or grippe).tw. (174088)
3 exp Influenza A virus/ (67327)
4 H1N1.tw. (29133)
5 or/1-4 (197774)
6 exp pregnancy/ (1384516)
7 exp Pregnancy Complications/ (464489)
8 exp Pregnancy Trimesters/ (680225)
9 Pregnant Women/ (48512)
10 pregnan*.tw. (900927)
11 (prenatal* or antenatal* or ante natal* or antepartum or ante partum or perinatal* or peri natal* or peripartum or peri partum).tw. (337753)
12 maternal*.tw. (414284)
13 exp Fetus/ (317744)
14 (fetus* or fetal* of foetus* or foetal*).tw. (241134)
15 (fetomaternal* or feto-maternal* or foetomaternal* or foeto-maternal*).tw. (6838)
16 Infectious Disease Transmission, Vertical/ (23258)
17 (vertical* adj3 transmi*).tw. (12683)
18 (intrauterine or intra-uterine or "in utero").tw. (152455)
19 (transplacent* or trans-placent* or uteroplacent* or utero-placent*).tw. (19347)
20 Prenatal Exposure Delayed Effects/ (38591)
21 exp Prenatal Diagnosis/ (145558)
22 or/6-21 (2173763)
23 5 and 22 (7024)
24 exp Obstetric Labor, Premature/ (50483)
25 ((labor* or labour* or birth* or deliver*) adj3 (preterm* or pre-term* or prematur* or pre-matur*)).tw. (81090)
26 (PTL or sPTL or PTB or sPTB or PTD or sPTD).tw. (13007)
27 Uterine Contraction/ (16575)
28 ((uterus* or uterine) adj3 contraction*).tw. (10403)
29 Cervical Ripening/ (2669)
30 ((cervical* or cervix*) adj3 (dilat* or ripen*)).tw. (10237)
31 exp Labor Onset/ (5165)
32 (preterm* or pre-term* or prematur* or pre-matur*).tw. (350831)
33 (27 or 28 or 29 or 30 or 31) and 32 (5480)
34 24 or 25 or 26 or 33 (105871)
35 exp Abortion, Spontaneous/ (57877)
36 miscarr*.tw. (23901)
37 (abort* adj3 (spontaneous* or habitual* or frequen* or recurr* or tubal)).tw. (27373)
38 exp fetal death/ (58308)
39 ((foetal or fetal or fetus* or foetus* or prenatal* or pre-natal* or perinatal* or peri-natal*) adj3 (death* or loss* or mortalit*)).tw. (64267)
40 Stillbirth/ (14133)

41 (stillbirth* or still-birth* or stillborn* or still-born*).tw. (27751)
42 or/35-41 (182123)
43 exp Infant, Low Birth Weight/ (69111)
44 SGA.tw. (11957)
45 (small* adj3 gestational age).tw. (15214)
46 Fetal Growth Retardation/ (32445)
47 ((fetus* or fetal* or foetus* or foetal* or intrauterine or intra-uterine) adj3 (growth restrict* or growth retard*)).tw. (32089)
48 IUGR.tw. (10253)
49 or/43-48 (117156)
50 Birth Weight/ (83654)
51 (birthweight or (weight adj3 (birth or neonat* or newborn*))).tw. (129904)
52 or/50-51 (162666)
53 (ae or co or de).fs. (7664156)
54 (safe or safety or side effect* or undesirable effect* or treatment emergent or tolerability or toxicity or adrs).ti,ab. (2113619)
55 (adverse adj2 (effect or effects or reaction or reactions or event or events or outcome or outcomes)).ti,ab. (637182)
56 ((birth* or fetus* or fetal* or foetus* or foetal* or neonat* or newborn* or pregnan*) adj3 outcome*).ti,ab. (86710)
57 or/53-56 (9322717)
58 23 and (34 or 42 or 49 or 52 or 57) (3009)
59 exp Animals/ not (exp Animals/ and Humans/) (8931926)
60 58 not 59 (2861)
61 (comment or editorial or interview or news).pt. (1533618)
62 (letter not (letter and randomized controlled trial)).pt. (1744241)
63 60 not (61 or 62) (2712)
64 63 use prmz (1162) [MEDLINE RECORDS]
65 influenza/ (91779)
66 exp influenza A/ (16797)
67 pandemic influenza/ (3420)
68 seasonal influenza/ (2991)
69 (influenza or flu or grippe).tw. (174088)
70 H1N1.tw. (29133)
71 or/65-70 (196367)
72 exp pregnancy/ (1384516)
73 exp pregnancy disorder/ (465452)
74 pregnant woman/ (48512)
75 pregnan*.tw. (900927)
76 (prenatal* or antenatal* or ante natal* or antepartum or ante partum or perinatal* or peri natal* or peripartum or peri partum).tw. (337753)
77 maternal*.tw. (414284)
78 fetus/ (246936)
79 (fetus* or fetal* of foetus* or foetal*).tw. (241134)
80 (fetomaternal* or feto-maternal* or foetomaternal* or foeto-maternal*).tw. (6838)
81 vertical transmission/ (23258)
82 (vertical* adj3 transmi*).tw. (12683)
83 (intrauterine or intra-uterine or "in utero").tw. (152455)

84 (transplacent* or trans-placent* or uteroplacent* or utero-placent*).tw. (19347)
85 prenatal exposure/ (38591)
86 exp prenatal diagnosis/ (145558)
87 or/72-86 (2234039)
88 71 and 87 (7272)
89 premature labor/ (43504)
90 ((labor* or labour* or birth* or deliver*) adj3 (preterm* or pre-term* or prematur* or pre-matur*)).tw. (81090)
91 (PTL or sPTL or PTB or sPTB or PTD or sPTD).tw. (13007)
92 uterus contraction/ (9479)
93 ((uterus* or uterine) adj3 contraction*).tw. (10403)
94 uterine cervix ripening/ (1853)
95 ((cervical or cervix*) adj3 (dilat* or ripen*)).tw. (10236)
96 Labor Onset/ (2598)
97 (preterm* or pre-term* or prematur* or pre-matur*).tw. (350831)
98 (92 or 93 or 94 or 95 or 96) and 97 (4801)
99 89 or 90 or 91 or 98 (103847)
100 spontaneous abortion/ (42829)
101 miscarr*.tw. (23901)
102 (abort* adj3 (spontaneous* or habitual* or frequen* or recurr* or tubal)).tw. (27373)
103 exp fetus death/ (34210)
104 ((foetal or fetal or fetus* or foetus* or prenatal* or pre-natal* or perinatal* or peri-natal*) adj3 (death* or loss* or mortalit*)).tw. (64267)
105 stillbirth/ (14133)
106 (stillbirth* or still-birth* or stillborn* or still-born*).tw. (27751)
107 or/100-106 (159124)
108 exp low birth weight/ (69111)
109 SGA.tw. (11957)
110 (small* adj3 gestational age).tw. (15214)
111 intrauterine growth retardation/ (32445)
112 ((fetus* or fetal* or foetus* or foetal* or intrauterine or intra-uterine) adj3 (growth restrict* or growth retard*)).tw. (32089)
113 IUGR.tw. (10253)
114 or/108-113 (117156)
115 birth weight/ (83654)
116 (birthweight or (weight adj3 (birth or neonat* or newborn*))).tw. (129904)
117 115 or 116 (162666)
118 fetus risk/ (2680)
119 (safe or safety or side effect* or undesirable effect* or treatment emergent or tolerability or toxicity or adrs).ti,ab. (2113619)
120 (adverse adj2 (effect or effects or reaction or reactions or event or events or outcome or outcomes)).ti,ab. (637182)
121 fetus outcome/ (7236)
122 pregnancy outcome/ (70113)
123 ((birth* or fetus* or fetal* or foetus* or foetal* or neonat* or newborn* or pregnan*) adj3 outcome*).ti,ab. (86710)
124 or/118-123 (2597023)
125 88 and (99 or 107 or 114 or 117 or 124) (2052)

126 exp animal experimentation/ or exp models animal/ or exp animal experiment/ or nonhuman/ or exp vertebrate/ (38785142)
127 exp humans/ or exp human experimentation/ or exp human experiment/ (29564179)
128 126 not 127 (9222634)
129 125 not 128 (1978)
130 letter.pt. (1748549)
131 randomized controlled trial/ (757592)
132 130 not (130 and 131) (1739911)
133 editorial.pt. (836788)
134 129 not (132 or 133) (1916)
135 134 use emczd (1253) [EMBASE RECORDS]
136 64 or 135 (2415) [MEDLINE AND EMBASE RECORDS]
137 remove duplicates from 136 (1839) [TOTAL UNIQUE HITS]
138 137 use prmz (1069) [MEDLINE UNIQUE HITS]
139 137 use emczd (770) [EMBASE UNIQUE HITS]

****
Final Strategies

Cochrane Library

Search Name: Influenza in Pregnancy - Fetal Effects / Outcomes (Post-PRESS)
Date Run: 05/12/14 04:30:51.158
Description: BORN (DF) - 2014 Dec 4

ID	Search	Hits
#1	[mh "Influenza, Human"]	1356
#2	(influenza or flu or grippe):ti,ab,kw	3962
#3	[mh "Influenza A virus"]	733
#4	H1N1:ti,ab,kw	675
#5	{or #1-#4}	3964
#6	[mh Pregnancy]	5824
#7	[mh "Pregnancy Complications"]	7834
#8	[mh "Pregnancy Trimesters"]	1474
#9	[mh "Pregnant Women"]	97
#10	pregnan*:ti,ab,kw	26229
#11	(prenatal* or antenatal* or ante natal* or antepartum or ante partum or perinatal* or peri natal* or peripartum or peri partum):ti,ab,kw	6379
#12	maternal*:ti,ab,kw	8535
#13	[mh Fetus]	1521
#14	(fetus* or fetal* of foetus* or foetal*):ti,ab,kw	2187
#15	(fetomaternal* or feto-maternal* or foetomaternal* or foeto-maternal*):ti,ab,kw	71
#16	[mh "Infectious Disease Transmission, Vertical"]	488
#17	(vertical* near/3 transmi*):ti,ab,kw	583
#18	(intrauterine or intra-uterine or "in utero"):ti,ab,kw	2773
#19	(transplacent* or trans-placent* or uteroplacent* or utero-placent*):ti,ab,kw	171
#20	[mh "Prenatal Exposure Delayed Effects"]	253
#21	[mh "Prenatal Diagnosis"]	938

#22 {or #6-#21} 32580
#23 #5 and #22 70
#24 [mh "Obstetric Labor, Premature"] 1117
#25 ((labor* or labour* or birth* or deliver*) near/3 (preterm* or pre-term* or prematur* or pre-matur*)):ti,ab,kw 3099
#26 (PTL or sPTL or PTB or sPTB or PTD or sPTD):ti,ab,kw 150
#27 [mh "Uterine Contraction"] 340
#28 ((uterus* or uterine) near/3 contraction*):ti,ab,kw 711
#29 [mh "Cervical Ripening"] 282
#30 ((cervical* or cervix*) near/3 (dilat* or ripen*)):ti,ab,kw 1588
#31 [mh "Labor Onset"] 507
#32 (preterm* or pre-term* or prematur* or pre-matur*):ti,ab,kw 13903
#33 (#27 or #28 or #29 or #30 or #31) and #32 282
#34 #24 or #25 or #26 or #33 3231
#35 [mh "Abortion, Spontaneous"] 688
#36 miscarr*:ti,ab,kw 700
#37 (abort* near/3 (spontaneous* or habitual* or frequen* or recurr* or tubal)):ti,ab,kw 892
#38 [mh "fetal death"] 208
#39 ((foetal or fetal or fetus* or foetus* or prenatal* or pre-natal* or perinatal* or peri-natal*) near/3 (death* or loss* or mortalit*)):ti,ab,kw 1059
#40 [mh Stillbirth] 51
#41 (stillbirth* or still-birth* or stillborn* or still-born*):ti,ab,kw 287
#42 {or #35-#41} 2477
#43 [mh "Infant, Low Birth Weight"] 1818
#44 SGA:ti,ab,kw 266
#45 (small* near/3 "gestational age"):ti,ab,kw 448
#46 [mh "Fetal Growth Retardation"] 274
#47 ((fetus* or fetal* or foetus* or foetal* or intrauterine or intra-uterine) near/3 (growth restrict* or growth retard*)):ti,ab,kw 522
#48 IUGR:ti,ab,kw 118
#49 {or #43-#48} 2548
#50 [mh "Birth Weight"] 1121
#51 (birthweight or (weight near/3 (birth or neonat* or newborn*))):ti,ab,kw 6072
#52 #50 or #51 6099
#53 (safe or safety or (side next effect*) or (undesirable next effect*) or "treatment emergent" or tolerability or toxicity or adrs):ti,ab,kw 138171
#54 (adverse near/2 (effect or effects or reaction or reactions or event or events or outcome or outcomes)):ti,ab,kw 73100
#55 Any MeSH descriptor with qualifier(s): [Adverse effects - AE, Complications - CO, Drug effects - DE] 205582
#56 ((birth* or fetus* or fetal* or foetus* or foetal* or neonat* or newborn* or pregnan*) near/3 outcome*):ti,ab,kw 6170
#57 {or #53-#56} 308149
#58 #23 and (#34 or #42 or #49 or #52 or #57) 38

DSR – 4
CENTRAL – 32
HTA – 1

NHS EED – 1

CINAHL Friday, December 05, 2014 1:27:43 PM

#	Query	Limiters/Expanders	Last Run Via	Results
S59	S23 AND S57	Limiters - Exclude MEDLINE records<br>Expanders - Apply related words<br>Search modes - Boolean/Phrase	Interface - EBSCOhost Research Databases<br>Search Screen - Advanced Search<br>Database - CINAHL	46
S58	S23 AND S57	Expanders - Apply related words<br>Search modes - Boolean/Phrase	Interface - EBSCOhost Research Databases<br>Search Screen - Advanced Search<br>Database - CINAHL	192
S57	S35 OR S42 OR S48 OR S51 OR S56	Expanders - Apply related words<br>Search modes - Boolean/Phrase	Interface - EBSCOhost Research Databases<br>Search Screen - Advanced Search<br>Database - CINAHL	163,231
S56	S52 OR S53 OR S54 OR S55	Expanders - Apply related words<br>Search modes - Boolean/Phrase	Interface - EBSCOhost Research Databases<br>Search Screen - Advanced Search<br>Database - CINAHL	144,054
S55	TI (((birth* or fetus* or fetal* or foetus* or foetal* or neonat* or newborn* or pregnan*) N3 outcome*)) OR AB (((birth* or fetus* or fetal* or foetus* or foetal* or neonat* or newborn* or	Expanders - Apply related words<br>Search modes - Boolean/Phrase	Interface - EBSCOhost Research Databases	7,769

	pregnan*) N3 outcome*))		Search Screen - Advanced Search Database - CINAHL	
S54	(MH "Pregnancy Outcomes")	Expanders - Apply related words Search modes - Boolean/Phrase	Interface - EBSCOhost Research Databases Search Screen - Advanced Search Database - CINAHL	10,330
S53	TI ((adverse N2 (effect or effects or reaction or reactions or event or events or outcome or outcomes))) OR AB ((adverse N2 (effect or effects or reaction or reactions or event or events or outcome or outcomes)))	Expanders - Apply related words Search modes - Boolean/Phrase	Interface - EBSCOhost Research Databases Search Screen - Advanced Search Database - CINAHL	36,939
S52	TI ((safe or safety or "side effect" or "side effects" or "undesirable effect" or "undersirable effects" or "treatment emergent" or tolerability or toxicity or adrs)) OR AB ((safe or safety or "side effect" or "side effects" or "undesirable effect" or "undersirable effects" or "treatment emergent" or tolerability or toxicity or adrs))	Expanders - Apply related words Search modes - Boolean/Phrase	Interface - EBSCOhost Research Databases Search Screen - Advanced Search Database - CINAHL	108,532
S51	S49 OR S50	Expanders - Apply related words Search modes - Boolean/Phrase	Interface - EBSCOhost Research Databases Search Screen - Advanced Search Database - CINAHL	11,305
S50	TI ((birthweight or (weight N3 (birth or neonat* or newborn*)))) OR AB ((birthweight or (weight N3 (birth or neonat* or newborn*))))	Expanders - Apply related words Search modes - Boolean/Phrase	Interface - EBSCOhost Research Databases	Display

			Search Screen - Advanced Search Database - CINAHL	
S49	(MH "Birth Weight")	Expanders - Apply related words Search modes - Boolean/Phrase	Interface - EBSCOhost Research Databases Search Screen - Advanced Search Database - CINAHL	Display
S48	S43 OR S44 OR S45 OR S46 OR S47	Expanders - Apply related words Search modes - Boolean/Phrase	Interface - EBSCOhost Research Databases Search Screen - Advanced Search Database - CINAHL	Display
S47	TI IUGR OR AB IUGR	Expanders - Apply related words Search modes - Boolean/Phrase	Interface - EBSCOhost Research Databases Search Screen - Advanced Search Database - CINAHL	Display
S46	TI (((fetus* or fetal* or foetus* or foetal* or intrauterine or intra-uterine) N3 (growth restrict* or growth retard*))) OR AB (((fetus* or fetal* or foetus* or foetal* or intrauterine or intra-uterine) N3 (growth restrict* or growth retard*)))	Expanders - Apply related words Search modes - Boolean/Phrase	Interface - EBSCOhost Research Databases Search Screen - Advanced Search Database - CINAHL	Display
S45	TI (small* N3 "gestational age") OR AB (small* N3 "gestational age")	Expanders - Apply related words Search modes - Boolean/Phrase	Interface - EBSCOhost Research Databases	Display

			Search Screen - Advanced Search Database - CINAHL	
S44	TI SGA OR AB SGA	Expanders - Apply related words Search modes - Boolean/Phrase	Interface - EBSCOhost Research Databases Search Screen - Advanced Search Database - CINAHL	Display
S43	(MH "Infant, Low Birth Weight+")	Expanders - Apply related words Search modes - Boolean/Phrase	Interface - EBSCOhost Research Databases Search Screen - Advanced Search Database - CINAHL	Display
S42	S36 OR S37 OR S38 OR S39 OR S40 OR S41	Expanders - Apply related words Search modes - Boolean/Phrase	Interface - EBSCOhost Research Databases Search Screen - Advanced Search Database - CINAHL	Display
S41	TI ((stillbirth* or still-birth* or stillborn* or still-born*)) OR AB ((stillbirth* or still-birth* or stillborn* or still-born*))	Expanders - Apply related words Search modes - Boolean/Phrase	Interface - EBSCOhost Research Databases Search Screen - Advanced Search Database - CINAHL	Display
S40	TI (((foetal or fetal or fetus* or foetus* or prenatal* or pre-natal* or perinatal* or peri-natal*) N3 (death* or loss* or mortalit*))) OR AB (((foetal or fetal or fetus* or foetus* or prenatal* or	Expanders - Apply related words Search modes - Boolean/Phrase	Interface - EBSCOhost Research Databases	Display

	pre-natal* or perinatal* or peri-natal*) N3 (death* or loss* or mortalit*)))		Search Screen - Advanced Search Database - CINAHL	
S39	(MH "Perinatal Death")	Expanders - Apply related words Search modes - Boolean/Phrase	Interface - EBSCOhost Research Databases Search Screen - Advanced Search Database - CINAHL	Display
S38	TI ((abort* N3 (spontaneous* or habitual* or frequen* or recurr* or tubal))) OR AB ((abort* N3 (spontaneous* or habitual* or frequen* or recurr* or tubal)))	Expanders - Apply related words Search modes - Boolean/Phrase	Interface - EBSCOhost Research Databases Search Screen - Advanced Search Database - CINAHL	Display
S37	TI miscarr* OR AB miscarr*	Expanders - Apply related words Search modes - Boolean/Phrase	Interface - EBSCOhost Research Databases Search Screen - Advanced Search Database - CINAHL	Display
S36	(MH "Abortion, Spontaneous+")	Expanders - Apply related words Search modes - Boolean/Phrase	Interface - EBSCOhost Research Databases Search Screen - Advanced Search Database - CINAHL	Display
S35	S24 OR S25 OR S26 OR S34	Expanders - Apply related words Search modes - Boolean/Phrase	Interface - EBSCOhost Research Databases	Display

			Search Screen - Advanced Search Database - CINAHL	
S34	S32 AND S33	Expanders - Apply related words Search modes - Boolean/Phrase	Interface - EBSCOhost Research Databases Search Screen - Advanced Search Database - CINAHL	Display
S33	S27 OR S28 OR S29 OR S30 OR S31	Expanders - Apply related words Search modes - Boolean/Phrase	Interface - EBSCOhost Research Databases Search Screen - Advanced Search Database - CINAHL	Display
S32	TI ((preterm* or pre-term* or prematur* or pre-matur*)) OR AB ((preterm* or pre-term* or prematur* or pre-matur*))	Expanders - Apply related words Search modes - Boolean/Phrase	Interface - EBSCOhost Research Databases Search Screen - Advanced Search Database - CINAHL	Display
S31	(MH "Labor Stage, First")	Expanders - Apply related words Search modes - Boolean/Phrase	Interface - EBSCOhost Research Databases Search Screen - Advanced Search Database - CINAHL	Display
S30	TI (((cervical* or cervix*) N3 (dilat* or ripen*))) OR AB (((cervical* or cervix*) N3 (dilat* or ripen*)))	Expanders - Apply related words Search modes - Boolean/Phrase	Interface - EBSCOhost Research Databases	Display

			Search Screen - Advanced Search Database - CINAHL	
S29	(MH "Cervix Dilatation and Effacement")	Expanders - Apply related words Search modes - Boolean/Phrase	Interface - EBSCOhost Research Databases Search Screen - Advanced Search Database - CINAHL	Display
S28	TI (((uterus* or uterine) N3 contraction*)) OR AB (((uterus* or uterine) N3 contraction*))	Expanders - Apply related words Search modes - Boolean/Phrase	Interface - EBSCOhost Research Databases Search Screen - Advanced Search Database - CINAHL	Display
S27	(MH "Uterine Contraction")	Expanders - Apply related words Search modes - Boolean/Phrase	Interface - EBSCOhost Research Databases Search Screen - Advanced Search Database - CINAHL	Display
S26	TI ((PTL or sPTL or PTB or sPTB or PTD or sPTD)) OR AB ((PTL or sPTL or PTB or sPTB or PTD or sPTD))	Expanders - Apply related words Search modes - Boolean/Phrase	Interface - EBSCOhost Research Databases Search Screen - Advanced Search Database - CINAHL	Display
S25	TI (((labor* or labour* or birth* or deliver*) N3 (preterm* or pre-term* or prematur* or pre-matur*))) OR AB (((labor* or labour* or birth* or deliver*) N3 (preterm* or pre-term* or prematur*	Expanders - Apply related words Search modes - Boolean/Phrase	Interface - EBSCOhost Research Databases	Display

	or pre-matur*)))		Search Screen - Advanced Search Database - CINAHL	
S24	(MH "Labor, Premature")	Expanders - Apply related words Search modes - Boolean/Phrase	Interface - EBSCOhost Research Databases Search Screen - Advanced Search Database - CINAHL	Display
S23	S5 AND S22	Expanders - Apply related words Search modes - Boolean/Phrase	Interface - EBSCOhost Research Databases Search Screen - Advanced Search Database - CINAHL	Display
S22	S6 OR S7 OR S8 OR S9 OR S10 OR S11 OR S12 OR S13 OR S14 OR S15 OR S16 OR S17 OR S18 OR S19 OR S20 OR S21	Expanders - Apply related words Search modes - Boolean/Phrase	Interface - EBSCOhost Research Databases Search Screen - Advanced Search Database - CINAHL	Display
S21	(MH "Prenatal Diagnosis+")	Expanders - Apply related words Search modes - Boolean/Phrase	Interface - EBSCOhost Research Databases Search Screen - Advanced Search Database - CINAHL	Display
S20	(MH "Prenatal Exposure Delayed Effects")	Expanders - Apply related words Search modes - Boolean/Phrase	Interface - EBSCOhost Research Databases	Display

			Search Screen - Advanced Search Database - CINAHL	
S19	TI ((transplacent* or trans-placent* or uteroplacent* or utero-placent*)) OR AB ((transplacent* or trans-placent* or uteroplacent* or utero-placent*))	Expanders - Apply related words Search modes - Boolean/Phrase	Interface - EBSCOhost Research Databases Search Screen - Advanced Search Database - CINAHL	Display
S18	TI ((intrauterine or intra-uterine or "in utero")) OR AB ((intrauterine or intra-uterine or "in utero"))	Expanders - Apply related words Search modes - Boolean/Phrase	Interface - EBSCOhost Research Databases Search Screen - Advanced Search Database - CINAHL	Display
S17	TI (vertical* n3 transmi*) OR AB (vertical* n3 transmi*)	Expanders - Apply related words Search modes - Boolean/Phrase	Interface - EBSCOhost Research Databases Search Screen - Advanced Search Database - CINAHL	Display
S16	(MH "Disease Transmission, Vertical")	Expanders - Apply related words Search modes - Boolean/Phrase	Interface - EBSCOhost Research Databases Search Screen - Advanced Search Database - CINAHL	Display
S15	TI ((fetomaternal* or feto-maternal* or foetomaternal* or foeto-maternal*)) OR AB ((fetomaternal* or feto-maternal* or foetomaternal* or foeto-maternal*))	Expanders - Apply related words Search modes - Boolean/Phrase	Interface - EBSCOhost Research Databases	Display

			Search Screen - Advanced Search Database - CINAHL	
S14	TI ((fetus* or fetal* of foetus* or foetal*)) OR AB ((fetus* or fetal* of foetus* or foetal*))	Expanders - Apply related words Search modes - Boolean/Phrase	Interface - EBSCOhost Research Databases Search Screen - Advanced Search Database - CINAHL	Display
S13	(MH "Fetus+")	Expanders - Apply related words Search modes - Boolean/Phrase	Interface - EBSCOhost Research Databases Search Screen - Advanced Search Database - CINAHL	Display
S12	TI maternal* OR AB maternal*	Expanders - Apply related words Search modes - Boolean/Phrase	Interface - EBSCOhost Research Databases Search Screen - Advanced Search Database - CINAHL	Display
S11	TI ((prenatal* or antenatal* or ante natal* or antepartum or ante partum or perinatal* or peri natal* or peripartum or peri partum)) OR AB ((prenatal* or antenatal* or ante natal* or antepartum or ante partum or perinatal* or peri natal* or peripartum or peri partum))	Expanders - Apply related words Search modes - Boolean/Phrase	Interface - EBSCOhost Research Databases Search Screen - Advanced Search Database - CINAHL	Display
S10	TI pregnan* OR AB pregnan*	Expanders - Apply related words Search modes - Boolean/Phrase	Interface - EBSCOhost Research Databases	Display

			Search Screen - Advanced Search Database - CINAHL	
S9	(MH "Expectant Mothers")	Expanders - Apply related words Search modes - Boolean/Phrase	Interface - EBSCOhost Research Databases Search Screen - Advanced Search Database - CINAHL	Display
S8	(MH "Pregnancy Trimesters+")	Expanders - Apply related words Search modes - Boolean/Phrase	Interface - EBSCOhost Research Databases Search Screen - Advanced Search Database - CINAHL	Display
S7	(MH "Pregnancy Complications+")	Expanders - Apply related words Search modes - Boolean/Phrase	Interface - EBSCOhost Research Databases Search Screen - Advanced Search Database - CINAHL	Display
S6	(MH "Pregnancy+")	Expanders - Apply related words Search modes - Boolean/Phrase	Interface - EBSCOhost Research Databases Search Screen - Advanced Search Database - CINAHL	Display
S5	S1 OR S2 OR S3 OR S4	Expanders - Apply related words Search modes - Boolean/Phrase	Interface - EBSCOhost Research Databases	Display

			Search Screen - Advanced Search Database - CINAHL	
S4	TI H1N1 OR AB H1N1	Expanders - Appliquer les mots connexes Search modes - Boolean/Phrase	Interface - EBSCOhost Research Databases Search Screen - Advanced Search Database - CINAHL	Display
S3	(MH "Influenza A Virus+")	Expanders - Appliquer les mots connexes Search modes - Boolean/Phrase	Interface - EBSCOhost Research Databases Search Screen - Advanced Search Database - CINAHL	Display
S2	TI ((influenza or flu or grippe)) OR AB ((influenza or flu or grippe))	Expanders - Appliquer les mots connexes Search modes - Boolean/Phrase	Interface - EBSCOhost Research Databases Search Screen - Advanced Search Database - CINAHL	Display
S1	(MH "Influenza, Human+")	Expanders - Appliquer les mots connexes Search modes - Boolean/Phrase	Interface - EBSCOhost Research Databases Search Screen - Advanced Search Database - CINAHL	Display
