# Supplementary material for: Maternal influenza and birth outcomes: systematic review of comparative studies
Source: BJOG. 2016 Jun 6;124(1):48–59. doi: 10.1111/1471-0528.14143 (PMC5216449; doi:10.1111/1471-0528.14143)
Supplement: Supplementary file 4 — Appendix S2. Risk of diagnostic ascertainment bias assessment [file BJO-124-48-s004.pdf]

Appendix S2. Risk of diagnostic ascertainment bias assessment

Rationale:
A general concern regarding ascertainment of clinical influenza illness among many studies in this review was that seeking medical attention for influenza illness, being selected for laboratory confirmatory testing for influenza infection, or being admitted to hospital could be motivated by concerns about the health of the pregnancy, not necessarily by the severity of influenza disease. If women at higher baseline risk for adverse pregnancy outcomes, or exhibiting signs of impending adverse outcomes, are differentially assessed and diagnosed with influenza, then exposure classification will be differential by outcome, thereby potentially inflating the magnitude of effect estimates. This potential for differential ascertainment of exposure was considered an important distinguishing feature of the eligible studies in this review. Since we were unaware of any formal tools to evaluate this potential bias, we developed a simple rating system specifically for this review.

Approach:
Two reviewers independently assessed the potential for diagnostic ascertainment bias in each study. We rated each study as having low, medium, high, or very high risk of diagnostic bias based on the method of exposure ascertainment (i.e., classification of subjects as having, or not having, clinical influenza disease and/or laboratory-confirmed influenza virus infection during pregnancy). All studies relying on medically-attended influenza (with or without laboratory confirmation) were considered medium risk or higher, depending on the clinical settings in which influenza was diagnosed (i.e., ambulatory versus hospitalizations) and whether these diagnoses were made anytime throughout gestation, or exclusively at the time of delivery (i.e., coincident diagnosis of influenza). Only studies with true prospective ascertainment of influenza were considered low risk. Descriptions of each category of risk are provided in the table below.

**Risk of diagnostic ascertainment bias**	**Description**
Low	▪ Prospective paired serology (i.e., maternal serum was routinely collected early in gestation and at the time of delivery, and the antibody titres were compared between the two samples)<br>▪ Prospective assessment collection of specimens from all women who develop symptoms of influenza-like illness throughout pregnancy (with laboratory testing of the specimens)
Medium	▪ Laboratory confirmation of influenza, carried out on symptomatic women in a range of clinical settings at any time throughout gestation [a]<br>▪ Medically-attended influenza, diagnosed in a range of clinical settings at any time throughout gestation [a]<br>▪ Routine prospective collection of self-reported influenza illness throughout pregnancy [b]
High [c]	▪ Medically-attended influenza, diagnosed only during hospitalizations ascertained at any time throughout gestation (i.e., during antepartum undelivered and delivery hospitalizations)<br>▪ Laboratory confirmation of influenza, carried out only on women who are severely ill or admitted to hospital<br>▪ Retrospective collection of self-reported influenza illness during pregnancy
Very high	▪ Medically-attended influenza, ascertained only at the time of the hospitalization to give birth (i.e., coincident temporal diagnosis of influenza)

[a] Can include hospitalizations in which influenza was diagnosed, as long as other ambulatory settings also were used to identify pregnant women with influenza.

[b] Although self-reported influenza would introduce other measurement inaccuracies, if measured prospectively the risk of differential reporting by outcome would be expected to be medium (not low, since women at risk of adverse outcome could still differentially report influenza exposure).
[c] We were particularly concerned about the potential during a hospitalization for women with complicated pregnancies (such as a preterm birth or threatened preterm birth), who would be subject to a higher level of diagnostic scrutiny than women with uncomplicated pregnancies, and hence a greater likelihood of diagnosis of influenza. If, however, it was clear that the major indication for the admission was the influenza (regardless of the pregnancy outcome), then the rating was changed to “medium risk”.
