# Supplementary material for: Maternal influenza and birth outcomes: systematic review of comparative studies
Source: BJOG. 2016 Jun 6;124(1):48–59. doi: 10.1111/1471-0528.14143 (PMC5216449; doi:10.1111/1471-0528.14143)
Supplement: Supplementary file 5 — Appendix S3. Assessment of primary study outcomes using adaptation of Grading of Recommendations Assessment, Development and Evaluation (GRADE) framework for assessing the quality of the evidence across studies [file BJO-124-48-s005.pdf]

Appendix S3. Assessment of primary study outcomes using adaptation of Grading of Recommendations Assessment, Development and Evaluation (GRADE) framework for assessing the quality of the evidence across studies

Preterm birth <37 weeks

**Profile of individual studies**		**Comments**
**Number of studies**<br>**Number of estimates**	16<br>17	▪ References: [1–16]<br>▪ There are 16 studies but 17 estimates because one study [7] reported separate estimates for 2009 A/pH1N1 and 2008–2009.<br>▪ Additional details on each study can be found in Table S4
**Estimated number of participants**	24,616,017	▪ Two studies[5,12] studies counted the total number of birth hospitalizations and the number of antenatal undelivered hospitalizations without reconciling admissions by unique pregnancy, so the counts are overestimated.
**Total number of preterm births**	1,207,209	▪ This is an underestimate since one study[12] did not provide information on the number of preterm births in their sample.
**Univariable results**		
Number of significant effect estimates > 1	2	
Number of non-significant effect estimates	9	
Number of significant effect estimates < 1	0	
Not reported	6	
**Multivariable results**		
Number of significant effect estimates > 1	4	▪ Point estimates ranged from 2.39 to 4.08
Number of non-significant effect estimates	9	▪ Point estimates ranging from 0.82 to 1.27
Number of significant effect estimates < 1	0	
Not reported	4	
**Newcastle Ottawa Score**		
0–5	0	
6–7	8	
8–9 (highest quality)	9	▪ The RCT[1] is listed with this category but was assessed using the Cochrane Collaboration tool for assessing risk of bias in randomized trials.[17] The risk of bias in the trial was rated as low.

**Risk of diagnostic ascertainment bias (S2 Appendix)**		
Very high	2	
High	8	
Medium	6	
Low	1	
**Statistical heterogeneity across studies: I^2=97%; 95% CI: 97–98**		
**GRADE assessment** [a,b]		**Comments**
**Phase of investigation**	Level 2 (high)	▪ We started by assigning a 'high' rating before applying other GRADE criteria since 15 of the 16 studies used cohort designs and sought to confirm the independent association between influenza during pregnancy and preterm birth, and the final study was a randomized clinical trial of influenza vaccination.[18]
**GRADE criteria (based on narrative review, not meta-analysis)**		
**Study limitations:**<br>▪ **Downgrade by -1** if most evidence is from studies with moderate or unclear risk of bias for most bias domains (serious limitations).<br>▪ **Downgrade by -2** if most evidence is from studies with high risk of bias for almost all bias domains (very serious limitations).	×	▪ Two studies had very high risk of diagnostic ascertainment bias (very serious limitations), and the rest were high or medium (serious limitations).<br>▪ **Downgrade by 2.**
**Inconsistency:** unexplained heterogeneity or variability in results across studies<br>▪ **Downgrade by -1** when estimates of the risk factor association with the outcome vary in direction (for example, some effects appear protective whereas others show risk) and the confidence intervals show no, or minimal overlap.	×	▪ See Forest plot. There is very high heterogeneity in results across studies, qualitatively and quantitatively (I^2=97%).<br>▪ The confidence intervals of the three studies with the large point estimates do not overlap with most of the null studies.<br>▪ The studies indicating a reduction in preterm birth risk all include the null value, and several are highly imprecise.<br>▪ **Downgrade by 1.**
**Indirectness:** the study sample, the prognostic factor, and/or the outcome in the primary studies do not accurately reflect the review question<br>▪ **Downgrade by -1** when: (1) the final sample only represents a subset of the population of interest; (2) when the complete breadth of the prognostic factor that is being considered in the review question is not well represented in the available studies; or (3) when the outcome that is being considered in the review question is not broadly represented.		▪ No change.

**Imprecision:**<br>▪ **Downgrade by -1** if the evidence is generated by a few studies involving a small number of participants and most of the studies provide imprecise results.<br>▪ For narrative summary: Within-study imprecision: (1) sample size justification is not provided and there are less than 10 outcome events for each prognostic variable (for dichotomous outcomes), and (2) no precision in the estimation of the effect size within each primary study. Across study imprecision: there are few studies and small number of participants across studies.		▪ No change.
**Publication bias:**<br>▪ **Downgrade by -1** unless the value of the risk/protective factor in predicting the outcome has been repetitively investigated, ideally by phase 2 and 3 studies.		▪ No change.
**Moderate/large effect size:**<br>▪ **Upgrade by +1** if moderate or large similar effect is reported by most studies.		▪ No change (most studies do not report large effect sizes and those that do have a high to very high risk of diagnostic ascertainment bias).
**Dose effect:**<br>▪ **Upgrade by +1** if possible gradient exists within and between primary studies.		▪ No change.
**GRADE: OVERALL QUALITY OF EVIDENCE (+, very low; ++, low; +++, moderate; ++++, high)**	**+ Very low**	

[a] Based on adaptation[18] of GRADE evaluation framework.[19]

[b] ╳ indicates serious limitations.

Small-for-gestational-age birth

Profile of individual studies		Comments
**Number of studies**<br>**Number of estimates**	5<br>6	▪ References: [4,16,7,10,20]<br>▪ Note there are 5 studies but 6 estimates because one study [7] reported an estimate for 2009 A/pH1N1 and one for the 2008–2009 influenza season.<br>▪ Additional details on each study can be found in Table S7
**Estimated number of participants**	245,127	
**Total number of small-for-gestational-age births**	20,748	▪ Estimated
**Univariable results**		
Number of significant effect estimates > 1	1	
Number of non-significant effect estimates	2	
Number of significant effect estimates < 1	0	
Not reported	3	
**Multivariable results**		
Number of significant effect estimates > 1	2	▪ Point estimates ranged from 1.59 to 1.66
Number of non-significant effect estimates	3	▪ Point estimates ranging from 0.71 to 1.14
Number of significant effect estimates < 1	0	
Not reported	1	
**Newcastle Ottawa Score**		
0–5	0	
6–7	1	
8–9 (highest quality)	5	
**Risk of diagnostic ascertainment bias (S2 Appendix)**		
Very high	0	
High	1	
Medium	4	
Low	1	
**Statistical heterogeneity across studies: I^2=43%; 95% CI: 0–76**		

GRADE assessment [a,b]		Comments
**Phase of investigation**	Level 2 (high)	▪ These cohort studies were considered to be Level 2 phase studies since they were seeking to confirm the independent association between influenza during pregnancy and small for gestational age.[18]<br>▪ Thus, the rating started off as 'high' before application of other GRADE criteria.[18]
**GRADE criteria (based on narrative review, not meta-analysis)**		
**<u>Study limitations</u>:**<br>▪ **Downgrade by -1** if most evidence is from studies with moderate or unclear risk of bias for most bias domains (serious limitations).<br>▪ **Downgrade by -2** if most evidence is from studies with high risk of bias for almost all bias domains (very serious limitations).	X	▪ **Downgrade by 1.**
**<u>Inconsistency</u>:** unexplained heterogeneity or variability in results across studies<br>▪ **Downgrade by -1** when estimates of the risk factor association with the outcome vary in direction (for example, some effects appear protective whereas others show risk) and the confidence intervals show no, or minimal overlap.		▪ No change.
**<u>Indirectness</u>:** the study sample, the prognostic factor, and/or the outcome in the primary studies do not accurately reflect the review question<br>▪ **Downgrade by -1** when: (1) the final sample only represents a subset of the population of interest; (2) when the complete breadth of the prognostic factor that is being considered in the review question is not well represented in the available studies; or (3) when the outcome that is being considered in the review question is not broadly represented.		▪ No change.
**<u>Imprecision</u>:**<br>▪ **Downgrade by -1** if the evidence is generated by a few studies involving a small number of participants and most of the studies provide imprecise results.<br>▪ For narrative summary: <u>Within-study imprecision</u>: (1) sample size justification is not provided and there are less than 10 outcome events for each prognostic variable (for dichotomous outcomes), and (2) no precision in the estimation of the effect size within each primary study. <u>Across study imprecision</u>: there are few studies and small number of participants across studies.		▪ No change.

**<u>Publication bias</u>:**<br>▪ **Downgrade by -1** unless the value of the risk/protective factor in predicting the outcome has been repetitively investigated, ideally by phase 2 and 3 studies.	╳	▪ **Downgrade by 1.**
**<u>Moderate/large effect size</u>:**<br>▪ **Upgrade by +1** if moderate or large similar effect is reported by most studies.		▪ No change.
**<u>Dose effect</u>:**<br>▪ **Upgrade by +1** if possible gradient exists within and between primary studies.		▪ No change.
**GRADE: OVERALL QUALITY OF EVIDENCE (+, very low; ++, low; +++, moderate; ++++, high)**	**++ Low**	

[a] Based on adaptation[18] of GRADE evaluation framework.[19]

[b] ╳ indicates serious limitations.

Fetal death

Profile of individual studies		Comments
**Number of studies**<br>**Number of estimates**	10<br>11	▪ References: [1,5,6,9,14,16,20–23]<br>▪ Note that one study [20] had no fetal death events among unexposed women (and only one event among exposed women), and two studies [1,16] reported two estimates, one for abortion, and the second for intrauterine fetal death (definitions were not provided).<br>▪ Additional details on each study can be found in Table S8
**Estimated number of participants**	17,666,768	▪ One study[5] study counted the total number of birth hospitalizations and the number of antenatal undelivered hospitalizations without reconciling admissions by unique pregnancy, so the counts are overestimated.
**Total number of fetal deaths**	103,902	▪ 7 out of the 10 studies had fewer than 20 fetal death outcomes in total (8,13,15,18–21)
**Univariable results**		
Number of significant effect estimates > 1	3	
Number of non-significant effect estimates	4	
Number of significant effect estimates < 1	0	
Not reported	4	
**Multivariable results**		
Number of significant effect estimates > 1	3	
Number of non-significant effect estimates	3	
Number of significant effect estimates < 1	0	
Not reported	5	
**Newcastle Ottawa Score**		
0–5	0	
6–7	6	
8–9 (highest quality)	5	▪ The RCT[1] is listed with this category but was assessed using the Cochrane Collaboration tool for assessing risk of bias in randomized trials.[17] The risk of bias in the trial was rated as low.

**Risk of diagnostic ascertainment bias (S2 Appendix)**		
Very high	1	
High	3	
Medium	2	
Low	4	
**Statistical heterogeneity across studies: I^2=41%; 95% CI: 0–69**		
**GRADE assessment** [a,b]		**Comments**
**Phase of investigation**	Level 2 (high)	▪ We started by assigning a 'high' rating before applying other GRADE criteria since 9 of the 10 studies used cohort designs and sought to confirm the independent association between influenza during pregnancy and fetal death, and the final study was a randomized clinical trial of influenza vaccination.[18]
**GRADE criteria (based on narrative review, not meta-analysis)**		
**<u>Study limitations</u>:**<br>▪ **Downgrade by -1** if most evidence is from studies with moderate or unclear risk of bias for most bias domains (serious limitations).<br>▪ **Downgrade by -2** if most evidence is from studies with high risk of bias for almost all bias domains (very serious limitations).	✕	▪ **Downgrade by 2.**<br>▪ Most evidence comes from one study[5] which is very large, but has a very high risk of diagnostic ascertainment bias.
**<u>Inconsistency</u>:** unexplained heterogeneity or variability in results across studies<br>▪ **Downgrade by -1** when estimates of the risk factor association with the outcome vary in direction (for example, some effects appear protective whereas others show risk) and the confidence intervals show no, or minimal overlap.		▪ No change.
**<u>Indirectness</u>:** the study sample, the prognostic factor, and/or the outcome in the primary studies do not accurately reflect the review question<br>▪ **Downgrade by -1** when: (1) the final sample only represents a subset of the population of interest; (2) when the complete breadth of the prognostic factor that is being considered in the review question is not well represented in the available studies; or (3) when the outcome that is being considered in the review question is not broadly represented.	✕	▪ **Downgrade by 1.**<br>▪ Studies used different definitions of fetal death, with differing gestational thresholds and many did not provide a definition.

**Imprecision:**<br>▪ **Downgrade by -1** if the evidence is generated by a few studies involving a small number of participants and most of the studies provide imprecise results.<br>▪ For narrative summary: Within-study imprecision: (1) sample size justification is not provided and there are less than 10 outcome events for each prognostic variable (for dichotomous outcomes), and (2) no precision in the estimation of the effect size within each primary study. Across study imprecision: there are few studies and small number of participants across studies.	✕	▪ **Downgrade by 1.**
**Publication bias:**<br>▪ **Downgrade by -1** unless the value of the risk/protective factor in predicting the outcome has been repetitively investigated, ideally by phase 2 and 3 studies.	✕	▪ **Downgrade by 1.**
**Moderate/large effect size:**<br>▪ **Upgrade by +1** if moderate or large similar effect is reported by most studies.		▪ No change.
**Dose effect:**<br>▪ **Upgrade by +1** if possible gradient exists within and between primary studies.		▪ No change.
**GRADE: OVERALL QUALITY OF EVIDENCE (+, very low; ++, low; +++, moderate; ++++, high)**	**+ Very low**	

[a] Based on adaptation[18] of GRADE evaluation framework.[19]

[b] ✕ indicates serious limitations.

References

1. Madhi SA, Cutland CL, Kuwanda L, Weinberg A, Hugo A, Jones S, et al. Influenza vaccination of pregnant women and protection of their infants. *N Engl J Med* 2014;371:918–31.

2. Ahrens KA, Louik C, Kerr S, Mitchell AA, Werler MM. Seasonal influenza vaccination during pregnancy and the risks of preterm delivery and small for gestational age birth. *Paediatr Perinat Epidemiol* 2014;28:498–509.

3. Doyle TJ, Goodin K, Hamilton JJ. Maternal and neonatal outcomes among pregnant women with 2009 pandemic influenza A(H1N1) illness in Florida, 2009-2010: a population-based cohort study. *PLoS One* 2013;8:e79040.

4. Naresh A, Fisher BM, Hoppe KK, Katov J, Xu J, Hart J, et al. A multicenter cohort study of pregnancy outcomes among women with laboratory-confirmed H1N1 influenza. *J Perinatol* 2013;33:939–43.

5. Martin A, Cox S, Jamieson DJ, Whiteman MK, Kulkarni A, Tepper NK. Respiratory illness hospitalizations among pregnant women during influenza season, 1998-2008. *Matern Child Health J* 2013;17:1325–31.

6. Håberg SE, Trogstad L, Gunnes N, Wilcox AJ, Gjessing HK, Samuelsen SO, et al. Risk of fetal death after pandemic influenza virus infection or vaccination. *N Engl J Med* 2013;368:333–40.

7. Hansen C, Desai S, Bredfeldt C, Cheetham C, Gallagher M, Li DK, et al. A large, population-based study of 2009 pandemic Influenza A virus subtype H1N1 infection diagnosis during pregnancy and outcomes for mothers and neonates. *J Infect Dis* 2012;206:1260–8.

8. Morken NH, Gunnes N, Magnus P, Jacobsson B. Risk of spontaneous preterm delivery in a low-risk population: the impact of maternal febrile episodes, urinary tract infection, pneumonia and ear-nose-throat infections. *Eur J Obstet Gynecol Reprod Biol* 2011;159:310–4.

9. Pierce M, Kurinczuk JJ, Spark P, Brocklehurst P, Knight M. Perinatal outcomes after maternal 2009/H1N1 infection: national cohort study. *BMJ* 2011;342:d3214.

10. McNeil SA, Dodds LA, Fell DB, Allen VM, Halperin BA, Steinhoff MC, et al. Effect of respiratory hospitalization during pregnancy on infant outcomes. *Am J Obstet Gynecol* 2011;204:S54–7.

11. Rogers VL, Sheffield JS, Roberts SW, McIntire DD, Luby JP, Trevino S, et al. Presentation of seasonal influenza A in pregnancy: 2003-2004 influenza season. *Obstet Gynecol* 2010;115:924–9.

12. Cox S, Posner SF, McPheeters M, Jamieson DJ, Kourtis AP, Meikle S. Hospitalizations with respiratory illness among pregnant women during influenza season. *Obstet Gynecol* 2006;107:1315–22.

13. Acs N, Bánhidy F, Puhó E, Czeizel AE. Pregnancy complications and delivery outcomes of pregnant women with influenza. *J Matern Fetal Neonatal Med* 2006;19:135–40.

14. Hartert TV, Neuzil KM, Shintani AK, Mitchell EF, Snowdon MS, Wood LB, et al. Maternal morbidity and perinatal outcomes among pregnant women with respiratory hospitalizations during influenza season. *Am J Obstet Gynecol* 2003;189:1705–12.

15. Tuyishime JD, De Wals P, Moutquin JM, Frost E. Influenza-like illness during pregnancy: results from a study in the eastern townships, Province of Quebec. *J Obstet Gynaecol Can* 2003;25:1020–5.

16. Nieto-Pascual L, Arjona-Berral JE, Marín-Martín EM, Muñoz-Gomariz E, Ilich I, Castelo-Branco C. Early prophylactic treatment in pregnant women during the 2009-2010 H1N1 pandemic: obstetric and neonatal outcomes. *J Obstet Gynaecol* 2013;33:128–4.

17. Higgins JPT, Altman DG, Gøtzsche PC, Juni P, Moher D, Oxman AD, et al. The Cochrane Collaboration's tool for assessing risk of bias in randomised trials. *BMJ* 2011;343:d5928.

18. Huguet A, Hayden JA, Stinson J, McGrath PJ, Chambers CT, Tougas ME, et al. Judging the quality of evidence in reviews of prognostic factor research: adapting the GRADE framework. *Syst Rev* 2013;2:71.

19. Guyatt G, Oxman AD, Akl EA, Kunz R, Vist G, Brozek J, et al. GRADE guidelines: 1. Introduction-GRADE evidence profiles and summary of findings tables. *J Clin Epidemiol* 2011;64:383–94.

20. Irving WL, James DK, Stephenson T, Laing P, Jameson C, Oxford JS, et al. Influenza virus infection in the second and third trimesters of pregnancy: a clinical and seroepidemiological study. *BJOG* 2000;107:1282–9.

21. Stanwell-Smith R, Parker AM, Chakraverty P, Soltanpoor N, Simpson CN. Possible association of influenza A with fetal loss: investigation of a cluster of spontaneous abortions and stillbirths. *Commun Dis Rep CDR Rev* 1994;4:R28–32.

22. Korones SB, Todaro J, Roane JA, Sever JL. Maternal virus infection after the first trimester of pregnancy and status of offspring to 4 years of age in a predominantly Negro population. *J Pediatr* 1970;77:245–51.

23. Wilson MG, Stein AM. Teratogenic effects of asian influenza. An extended study. *JAMA* 1969;210:336–7.
