# Supplementary material for: Maternal influenza and birth outcomes: systematic review of comparative studies
Source: BJOG. 2016 Jun 6;124(1):48–59. doi: 10.1111/1471-0528.14143 (PMC5216449; doi:10.1111/1471-0528.14143)
Supplement: Supplementary file 6 — Appendix S4. Secondary outcomes [file BJO-124-48-s006.pdf]

Appendix S4. Secondary outcomes

Three studies investigated preterm birth at earlier gestational age thresholds (Table S9 and Table S10).[1–3] Two studies[1,2] reported adjusted odds ratios from these analyses that were higher in magnitude than their conventional preterm birth analyses (based on the cut-off of <37 weeks). Mean gestational age comparisons were reported by six studies (Table S11). Apart from one study that reported an unadjusted mean difference of one full week between women who had 2009 pandemic H1N1 influenza disease during pregnancy compared with those who did not (mean gestational age 37.3 weeks and 38.3 weeks, respectively; *P*-value <0.001),[1] mean differences reported by other studies were small (between 0.1 and 0.3 weeks) and not statistically significant.

Ten of 12 estimates for low birth weight (<2,500 grams) had confidence intervals that included the null value (point estimates ranged from 0.40 to 1.48) (Table S12). Two studies from the 2009 H1N1 pandemic report statistically significant odds ratios greater than one (adjusted ORs ranged from 1.67 to 3.2);[1,2] however, neither accounted for gestational age in the analysis of low birth weight, despite a higher risk of preterm birth among women with influenza illness (approximately 24% among women with pandemic H1N1 illness in both studies).[1,2] One study reported that a significant birth weight reduction (by -255 grams) among women with severe pandemic 2009 H1N1 illness, compared with women from the general obstetrical population, disappeared following adjustment for gestational age.[2] In contrast, two other studies from the 2009 pandemic reported no association between maternal influenza disease and low birth weight, but both ascertained a lower severity of maternal influenza illness and both accounted for gestational age in their analyses.[4,5] No difference in the proportion of low birth weight infants between the active treatment and placebo groups was observed in the RCT.[6]

The results from 13 studies that reported continuous birth weight are challenging to interpret, owing to inconsistent reporting of absolute differences and variance measures, both of which were computed by review authors where possible (Table S13). Of four studies that accounted for gestational age either through restriction to term births or matching on gestational age, two reported a higher mean birth weight[7,8] and two a lower mean birth weight[9,10] among infants born to women with influenza illness or infection during pregnancy compared with no influenza (mean difference ranged from -99 grams to 70 grams; Table S13). Among studies that did not account for gestational age in any way, both reductions and increases in birth weight were reported. The median birth weight did not differ between randomized treatment groups in the RCT.[6]

References

1. Doyle TJ, Goodin K, Hamilton JJ. Maternal and neonatal outcomes among pregnant women with 2009 pandemic influenza A(H1N1) illness in Florida, 2009-2010: a population-based cohort study. *PLoS One* 2013;8:e79040.

2. Pierce M, Kurinczuk JJ, Spark P, Brocklehurst P, Knight M. Perinatal outcomes after maternal 2009/H1N1 infection: national cohort study. *BMJ* 2011;342:d3214.

3. Rogers VL, Sheffield JS, Roberts SW, McIntire DD, Luby JP, Trevino S, et al. Presentation of seasonal influenza A in pregnancy: 2003-2004 influenza season. *Obstet Gynecol* 2010;115:924–9.

4. Håberg SE, Trogstad L, Gunnes N, Wilcox AJ, Gjessing HK, Samuelsen SO, et al. Risk of fetal death after pandemic influenza virus infection or vaccination. *N Engl J Med* 2013;368:333–40.

5. Hansen C, Desai S, Bredfeldt C, Cheetham C, Gallagher M, Li DK, et al. A large, population-based study of 2009 pandemic Influenza A virus subtype H1N1 infection diagnosis during pregnancy and outcomes for mothers and neonates. *J Infect Dis* 2012;206:1260–8.

6. Madhi SA, Cutland CL, Kuwanda L, Weinberg A, Hugo A, Jones S, et al. Influenza vaccination of pregnant women and protection of their infants. *N Engl J Med* 2014;371:918–31.

7. Acs N, Bánhidy F, Puhó E, Czeizel AE. Pregnancy complications and delivery outcomes of pregnant women with influenza. *J Matern Fetal Neonatal Med* 2006;19:135–40.

8. Irving WL, James DK, Stephenson T, Laing P, Jameson C, Oxford JS, et al. Influenza virus infection in the second and third trimesters of pregnancy: a clinical and seroepidemiological study. *BJOG* 2000;107:1282–9.

9. McNeil SA, Dodds LA, Fell DB, Allen VM, Halperin BA, Steinhoff MC, et al. Effect of respiratory hospitalization during pregnancy on infant outcomes. *Am J Obstet Gynecol* 2011;204:S54–7.

10. Hartert TV, Neuzil KM, Shintani AK, Mitchell EF, Snowdon MS, Wood LB, et al. Maternal morbidity and perinatal outcomes among pregnant women with respiratory hospitalizations during influenza season. *Am J Obstet Gynecol* 2003;189:1705–12.
